# Relative quantification of proteins and post-translational modifications in proteomic experiments with shared peptides: a weight-based approach

**DOI:** 10.1093/bioinformatics/btaf046

**Published:** 2025-01-31

**Authors:** Mateusz Staniak, Ting Huang, Amanda M Figueroa-Navedo, Devon Kohler, Meena Choi, Trent Hinkle, Tracy Kleinheinz, Robert Blake, Christopher M Rose, Yingrong Xu, Pierre M Jean Beltran, Liang Xue, Małgorzata Bogdan, Olga Vitek

**Affiliations:** Faculty of Mathematics and Computer Science, University of Wrocław, Wrocław, 50-383, Poland; Centre for Statistics, Hasselt University, Diepenbeek, 3590, Belgium; Khoury College of Computer Sciences, Northeastern University, Boston, MA, 02115, United States; Barnett Institute of Chemical and Biological Analysis, Northeastern University, Boston, MA, 02115, United States; Barnett Institute of Chemical and Biological Analysis, Northeastern University, Boston, MA, 02115, United States; Khoury College of Computer Sciences, Northeastern University, Boston, MA, 02115, United States; Barnett Institute of Chemical and Biological Analysis, Northeastern University, Boston, MA, 02115, United States; Department of Microchemistry, Proteomics, and Lipidomics, Genentech, Inc., South San Francisco, CA, 94080, United States; Department of Microchemistry, Proteomics, and Lipidomics, Genentech, Inc., South San Francisco, CA, 94080, United States; Department of Biochemical and Cellular Pharmacology, Genentech, Inc., South San Francisco, CA, 94080, United States; Department of Biochemical and Cellular Pharmacology, Genentech, Inc., South San Francisco, CA, 94080, United States; Department of Microchemistry, Proteomics, and Lipidomics, Genentech, Inc., South San Francisco, CA, 94080, United States; Discovery Sciences, Pfizer Inc., Groton, CT, 06340, United States; Machine Learning and Computational Sciences, Pfizer Inc., Cambridge, MA, 02139, United States; Machine Learning and Computational Sciences, Pfizer Inc., Cambridge, MA, 02139, United States; Faculty of Mathematics and Computer Science, University of Wrocław, Wrocław, 50-383, Poland; Khoury College of Computer Sciences, Northeastern University, Boston, MA, 02115, United States; Barnett Institute of Chemical and Biological Analysis, Northeastern University, Boston, MA, 02115, United States

## Abstract

**Motivation:**

Bottom-up mass spectrometry-based proteomics studies changes in protein abundance and structure across conditions. Since the currency of these experiments are peptides, i.e. subsets of protein sequences that carry the quantitative information, conclusions at a different level must be computationally inferred. The inference is particularly challenging in situations where the peptides are shared by multiple proteins or post-translational modifications. While many approaches infer the underlying abundances from unique peptides, there is a need to distinguish the quantitative patterns when peptides are shared.

**Results:**

We propose a statistical approach for estimating protein abundances, as well as site occupancies of post-translational modifications, based on quantitative information from shared peptides. The approach treats the quantitative patterns of shared peptides as convex combinations of abundances of individual proteins or modification sites, and estimates the abundance of each source in a sample together with the weights of the combination. In simulation-based evaluations, the proposed approach improved the precision of estimated fold changes between conditions. We further demonstrated the practical utility of the approach in experiments with diverse biological objectives, ranging from protein degradation and thermal proteome stability, to changes in protein post-translational modifications.

**Availability and implementation:**

The approach is implemented in an open-source R package MSstatsWeightedSummary. The package is currently available at https://github.com/Vitek-Lab/MSstatsWeightedSummary (doi: 10.5281/zenodo.14662989). Code required to reproduce the results presented in this article can be found in a repository https://github.com/mstaniak/MWS_reproduction (doi: 10.5281/zenodo.14656053).

## 1 Introduction

Mass spectrometry (MS)-based proteome profiling experiments characterize protein composition of complex biological mixtures (Aebersold and Mann 2016, Miller and Smith 2023). They determine changes in protein abundance and structure across conditions (such as treatments) that are more systematic than as expected by random chance (Lin *et al.* 2022).

In bottom-up proteomics, proteins are enzymatically digested into peptides. Some experiments label peptides from up to 18 samples, e.g. using tandem mass tags (TMT), and combine them into a single mixture called plex (Thompson *et al.* 2003, Sivanich *et al.* 2022). Each TMT label forms a channel, where the intensity is informative of the abundance of the peptide in the original sample. The peptides in a mixture are subsequently ionized and subjected to mass analysis, producing features in mass spectra. Computational tools such as MaxQuant (Tyanova *et al.* 2016), Proteome Discoverer (Orsburn 2021), or many others identify the peptide ions underlying the spectral features in terms of their amino acid sequence, and quantify their abundance.

The peptide ions are the main currency of these experiments, and carry the most direct quantitative information. However, in applications such as MS-based drug development, the scientific question focuses not on the peptide ions but on proteins targeted by therapeutics (Macklin *et al.* 2020). For example, degradation studies characterize changes in overall protein abundance over time (Békés *et al.* 2022), as they seek to affect functions of proteins. Similarly, thermal profiling experiments (Kurzawa *et al.* 2023) aim to characterize protein drug targets by monitoring changes in thermal stability of proteins. Ideally, these applications would distinguish protein isoforms, as well as proteins resulting from events such as alternative splicing. However, characterizing such proteins is challenging, as they have high sequence similarity, and produce *shared peptides*, i.e. peptides whose amino acid sequences match multiple proteins. The shared peptides can constitute over 50% of all the possible peptides in the experiment, when all such events are considered, as shown in Schork *et al.* (2022), Madhira (2016), and Wilmarth (2020) (https://pwilmart.github.io/blog/2020/09/19/shotgun-quantification-part2).

Since proteins are not observed directly, their identity and abundance are computationally inferred. The inference involves two aspects. The first aspect is identifying the protein sequence from the observed peptides, referred to as the protein inference problem (Nesvizhskii and Aebersold 2005). A second aspect of computational inference is inference of protein abundance, i.e. summarizing the quantitative information in the peptide ions into a single quantity per protein per sample per run (Kohler *et al.* 2023a). Proteins with similar but distinct sequences may differ significantly in how their abundances change between treatments or conditions (Bludau *et al.* 2021, Plubell *et al.* 2022, Kurzawa *et al.* 2023).

There is currently no generally accepted strategy for estimating the abundance of proteins in presence of shared peptides (Bludau *et al.* 2021), and most methods quantify individual proteins or protein groups based on unique peptides (Goeminne *et al.* 2018; Dermit *et al.*, 2021, Kohler *et al.* 2023a). Some authors advocate forgoing protein-level summarization altogether, and proceed with peptide-level statistical analysis (Plubell *et al.* 2022). Alternatively, Triqler (Truong *et al.* 2023) advocates for an integrated approach that combines identification and quantification, however it also assumes the use of unique peptides. Overall, these approaches do not provide a sufficient quantitative insight into protein isoforms, or proteins resulting from alternative splicing.

The challenge of inference of identity and abundance of analytes from shared sequence information extends beyond inference of protein abundance. A conceptually similar problem arises in studies focusing on protein post-translational modifications (PTMs). Upon digestion, multiple peptides can carry a same modification site, and a peptide can carry multiple modifications. Therefore, the occupancy of a modification site must also be computationally inferred. Similarly to protein-level summarization, there is no generally accepted strategy for estimating site occupancy of PTMs with shared peptides.

This article addresses the limitations of the methods above. We propose a statistical approach that models the quantitative profiles of shared peptides in biological samples as convex combinations of the profiles of their sources. It estimates the relative abundance of each protein or PTM site, together with their weights. The approach is implemented in an open-source R package MSstatsWeightedSummary compatible with the workflow of R/Bioconductor package MSstatsTMT (Huang *et al.* 2020). Currently, the approach is applicable to experiments with isobaric labeling.

### 1.1 Background

Below, we first review the existing methods relevant for detecting differentially abundant proteins in the presence of shared peptides. We distinguish three steps of differential analysis: protein inference, protein-level summarization, and statistical modeling of summarized proteins for relative abundance estimation. We then highlight the similarities and the differences of detecting differentially abundant proteins and detecting differentially abundant PTMs.

#### 1.1.1 Protein inference based on amino acid sequences

**Figure 1.**
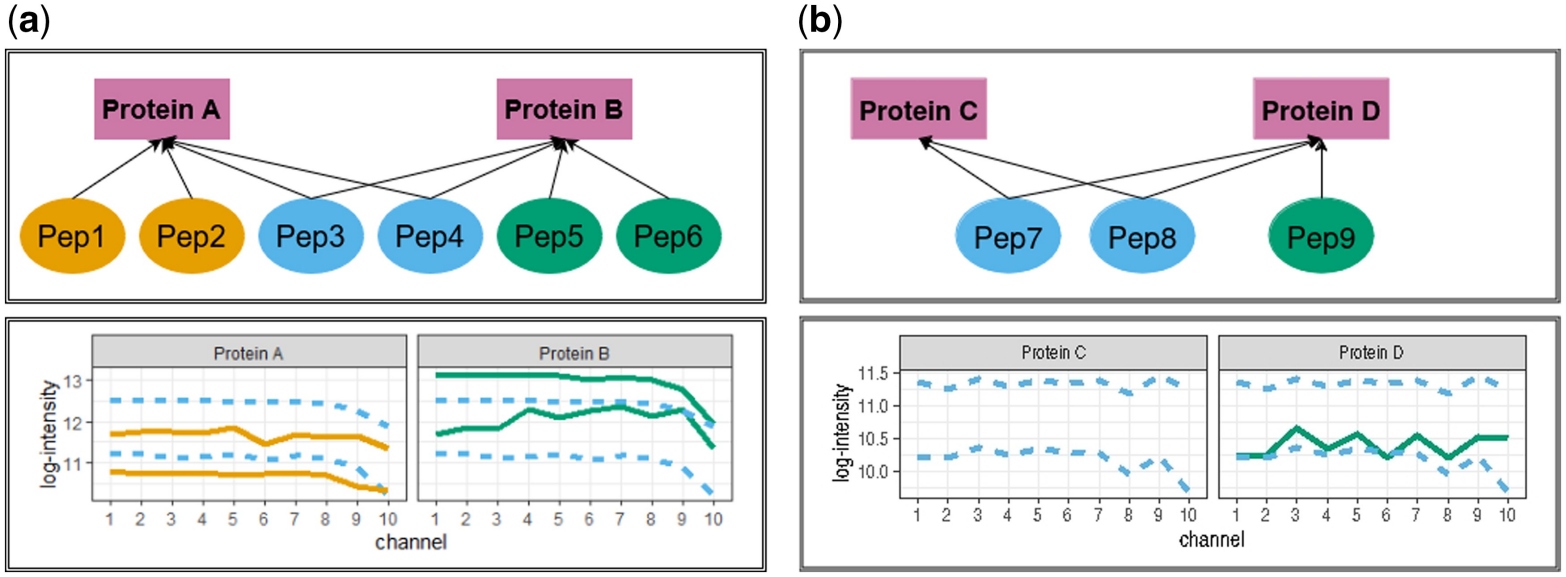
Simple examples of protein inference. Rectangles are proteins, ovals are peptides. Arrows indicate protein membership of a peptide based on the amino acid sequence information. Peptides 1, 2, 5, 6, and 9 are unique, while peptides 3, 4, 7, and 8 are shared. Quantitative profiles of each peptide across biological samples (allocated to TMT channels) are shown below the peptide–protein graphs. Solid lines indicate unique peptides, dashed lines indicate shared peptides. (a) Proteins A and B have unique peptides, and share two peptides. (b) Proteins C and D share two peptides, but only D has a unique peptide.

Figure 1 illustrates the problem of protein inference in presence of shared peptides via bipartite graphs. In (a), proteins A and B share peptides with each other, but also have unique peptides. In (b), proteins C and D also share peptides, however only D has a unique peptide. Since proteins in Fig. 1a and b do not share peptides, they can be analyzed separately. From the graph theory perspective, the two groups of proteins form distinct connected sub-graphs, which we will refer to as ‘protein clusters’.

The *et al.* (2018) distinguished three approaches for inferring protein identities in the presence of shared peptides: exclusion, inclusion, and parsimony. Exclusion removes all the shared peptides from the analysis. One example of this approach is in Savitski *et al.* (2015). In Fig. 1b, this results in a loss of protein C. This criterion is often used even more stringently, removing proteins with only one unique peptide, and losing both proteins C and D. This principle is known as a two-peptide rule (Serang *et al.* 2012). Inclusion assigns every shared peptide to each matching protein. For example, in Fig. 1a, protein A is characterized by peptides 1, 2, 3, and 4, and protein B is characterized by peptides 3, 4, 5, and 6. With this approach no protein is lost, and the abundances of peptides 3 and 4 are attributed in equal measure to both proteins, leading to potential quantitative bias. ProteinProphet (Nesvizhskii *et al.* 2003) is one example of this approach. Finally, parsimony aims to find a minimal set of proteins that explains the presence of all the identified peptides. In Fig. 1b, this either excludes protein C, or leads to a new protein {C, D}. Similarly to inclusion, parsimony can group into a same protein peptides with different quantitative patterns. Parsimony is most commonly used, and implemented in popular signal processing tools such as MaxQuant (Tyanova *et al.* 2016) and Proteome Discoverer (Orsburn 2021).

#### 1.1.2 Protein inference assisted by peptide abundance

While most protein inference algorithms rely on the amino acid information (Huang *et al.* 2012), several recent approaches advocated for using quantitative information: Quantifere (Lukasse and America 2014), PeCorA [Peptide Correlation Analysis (Dermit *et al.* 2021)], COPF [COrrelation-based functional ProteoForm assessment (Bludau *et al.* 2021)], VIQoR (Tsiamis and Schw Mmle 2022). We provide additional information about these methods in Supplementary Section S1.1.

#### 1.1.3 Statistical framework for protein summarization

Most protein inference methods output proteins or groups of proteins under a common label (protein groups), together with their peptides. When the goal of the experiment is to detect changes in protein abundance, the step after protein inference aggregates the abundances of peptides into a single quantity per protein (or protein group) per biological sample, comparable between treatments or conditions.

Multiple methods for protein summarization exist, ranging from simple means or medians of peptide abundances in each sample, to model-based approaches such Triqler (Truong *et al.* 2023) and MSstats (Kohler *et al.* 2023a). Other approaches such as MSqRob (Sticker *et al.* 2020) work directly with peptide-level data. All these methods assume that proteins or protein groups are characterized by uniquely matched peptides. Below we describe the statistical framework for protein-level summarization of experiments with TMT labeling in the open-source software MSstatsTMT (Huang *et al.* 2020).

Consider a protein (or a protein group) characterized by f=1,…,F spectral features, i.e. peptide ions matched to that protein or group. The experiment profiles b=1,…,B biological samples from each of g=1,…,G groups (also called conditions), in c=1,…,C channels for each of m=1,…,M mixtures. For simplicity, we assume that the experiment has no technical replicates. Experiments with M>1 mixtures typically dedicate one channel per mixture for reference material used for normalization (Plubell *et al.* 2017). For the purposes of protein summarization, MSstatsTMT models each protein and each mixture separately with a linear model

(1) $$\begin{array}{c} X_{fc}=\mu +\mathrm{Featur}e_{f}+\mathrm{Channe}l_{c}+\varepsilon_{fc},\\ \sum_{f=1}^{F}Fe\mathrm{atur}e_{f}=0,\sum_{c=1}^{C}Ch\mathrm{anne}l_{c}=0,\;\varepsilon_{fc}\;\overset{\mathrm{iid}}{∼}\;\cdot (0,\sigma_{\varepsilon }^{2}), \end{array}$$

where $X_{fc}$ denotes the observed ${\;\text{log}\;}_{2}$-intensity of feature f in channel c, μ denotes the overall mean protein abundance, $\mathrm{Featur}e_{f}$ and $\mathrm{Channe}l_{c}$ denote the additive main effects of feature f and channel c, and $\varepsilon_{fc}$ denotes independent, identically distributed and non-systematic noise. The model is linear in parameters μ, $\mathrm{Featur}e_{f}$, and $\mathrm{Channe}l_{c}$. MSstatsTMT estimates the parameters using a robust Tukey Median Polish (TMP) (Kohler *et al.* 2023a) approach. Finally, the estimate of protein abundance in channel c is

(2) $$Y_{c}=\hat{\mu }+{\hat{\mathrm{Channel}}}_{c},\;c=1,\ldots ,C.$$

$Y_{c}$ serves as input to the downstream differential analysis. The indices of proteins and mixtures in Equations (1) and (2) are omitted for simplicity.

#### 1.1.4 Statistical framework for differential abundance

Once protein abundances are summarized, the next step specifies a statistical model for the protein-level summaries. Such a model characterizes the available sources of variation, and serves as a basis for tests for differential abundance. Many statistical models have been proposed, e.g. DeqMS (Zhu *et al.* 2020), MSqRob (Sticker *et al.* 2020), or MSstats (Kohler *et al.* 2023a). They were reviewed in detail in Bai *et al.* (2023). Below we describe MSstatsTMT, which flexibly accommodates diverse experimental designs in experiments with TMT labels (Huang *et al.* 2020, 2023). MSstatsTMT fits a separate linear model to each protein summary. For example, for group comparison designs, it fits

(3) $$Y_{\mathrm{gbm}}=\mu +\mathrm{Conditio}n_{g}+\mathrm{Mixtur}e_{m}+\varepsilon_{\mathrm{mgb}},$$

where $\sum_{g=1}^{G}\mathrm{Conditio}n_{g}=0$,

$$\mathrm{Mixtur}e_{m}\;\overset{\mathrm{iid}}{∼}\;N(0,\sigma_{M}^{2}),\;\text{and}\;\varepsilon_{\mathrm{gbm}}\;\overset{\mathrm{iid}}{∼}\;N(0,\sigma^{2}).$$

As another example, consider a more complex design that profiles biological replicates across multiple groups, collects repeated measurements on the biological replicates in time, and allocates measurements from each biological replicate to its own mixture. MSstatsTMT fits the model

(4) $$Y_{\mathrm{ctm}}=\mu +\mathrm{ConditionTim}e_{ct}+\mathrm{Mixtur}e_{m}+\varepsilon_{\mathrm{mct}},$$

where $\sum_{ct}Co\mathrm{nditionTim}e_{ct}=0$,

$$\mathrm{Mixtur}e_{m}\;\overset{\mathrm{iid}}{∼}\;N(0,\sigma_{M}^{2}),\;\varepsilon_{\mathrm{ctm}}\;\overset{\mathrm{iid}}{∼}\;N(0,\sigma^{2}).$$

In this notation, $\mathrm{ConditionTim}e_{ct}$ represents all the combinations of conditions and times, and *Mixture* is confounded with *Subject*. For each model and each protein, tests of differential abundance specify a null hypothesis, e.g. $H_{0}:\mathrm{Conditio}n_{c}=\mathrm{Conditio}n_{c^{\prime }}$ or $H_{0}:\mathrm{ConditionTim}e_{ct}=\mathrm{ConditionTim}e_{ct^{\prime }}$. All the model parameters are estimated using restricted maximum likelihood. The parameter estimates and their standard errors are combined into *t*-statistics to derive *P*-values, which in turn are adjusted to control false discovery rate (FDR).

#### 1.1.5 Relative PTM quantification

The goal of MS-based relative PTM quantification is to assess changes in occupancy of a PTM site across conditions, and to distinguish it from overall changes in protein abundance. Similarly to proteome profiling, this requires summarizing the quantitative information relevant to a PTM site over multiple peptides. The relationship between PTM sites and peptides is similar to the relationship between proteins and peptides in proteome profiling. Peptides with a unique modification are used directly to quantify the occupancy of the PTM site. Peptides with multiple modifications match different sites, similarly to shared peptides.

##### 1.1.5.1. PTM site summarization

Two methods have been recently proposed for PTM summarization, msqrob2PTM (Demeulemeester *et al.* 2024) and MSstatsPTM (Kohler *et al.* 2023b). msqrob2PTM repeatedly uses peptides with multiple modifications to quantify each PTM site, effectively implementing the inclusion approach from protein inference. In contrast, MSstatsPTM combines the two modification sites into an artificial site (called concatenation), and estimates a separate log-fold change for this combination, effectively implementing protein grouping.

##### 1.1.5.2. Statistical modeling and differential abundance

Similarly to proteome profiling, the next step is statistical modeling of the summarized abundances. To distinguish changes in PTM site occupancy from overall changes in protein abundance, msqrob2PTM normalizes feature-level ${\;\text{log}\;}_{2}$-intensities by subtracting the estimated abundance of the unmodified protein in the sample. The normalized feature intensities are then modeled with a robust linear model that accounts for differences between biological conditions. In contrast, MSstatsPTM separately summarizes the modified and the unmodified features corresponding to a PTM site with the MSstats workflow (Equation (1)). It then fits a separate protein-level model (e.g. Equations (3) and (4)) to each summary to reflect the experimental design. Finally, the null hypothesis compares changes in the expected abundance of the PTM site between conditions to the changes of the unmodified protein.

## 2 Materials and methods

We propose to extend protein summarization in MSstatsTMT (Equation (1)) for experiments with TMT labels to simultaneously estimate the abundances of proteins with shared peptides. Similarly to Quantifere, PeCorA, and COPF, we consider similarities between the feature-level profiles, however we do not attempt to cluster the profiles or assign them to an isoform. Similarly to VIQoR, we directly quantify the contribution of a peptide to protein-level summaries in the form of weights, however we output not log-fold changes but full protein-level summaries compatible with statistical modeling of various experimental designs.

### 2.1 Proposed statistical model

Following MSstatsTMT, we use the term *Feature* to describe a peptide ion, and denote the ${\;\text{log}\;}_{2}$-intensity of feature f in channel c by $X_{cf}$. We proceed with separate summarization for each TMT mixture (and omit the mixture indices for simplicity). Unlike MSstatsTMT, which summarizes one protein at a time, we simultaneously model a cluster of K>1 proteins that share peptides, such as in Fig. 1. For each spectral feature f, we define the set of protein memberships

V(f)={k∈1,…,K:feature f matches Protein k}

and extend the MSstatsTMT summarization model in Equation (1) as

$$X_{cf}=\mu +\sum_{k\in V(f)}We\mathrm{igh}t_{fk}\left(\mathrm{Protei}n_{k}+\mathrm{Channe}l_{kc}\right)$$

 

(5) $$+\mathrm{Featur}e_{f}+\varepsilon_{cf},\;\varepsilon_{fc}\;\overset{\mathrm{iid}}{∼}\cdot (0,\sigma_{\varepsilon }^{2})\;$$

under the typical linear model constraints

$$\begin{array}{c} \sum_{k=1}^{K}Pr\mathrm{otei}n_{k}=0,\;\forall_{k}\;\sum_{c=1}^{C}Ch\mathrm{anne}l_{kc}=0,\\ \sum_{f=1}^{F}Fe\mathrm{atur}e_{f}=0 \end{array}$$

and two new additional constraints

$$\forall_{f}\;\sum_{k\in V(f)}We\mathrm{igh}t_{fk}=1,\;\forall_{f,k}\;\mathrm{Weigh}t_{fk}\geq 0$$

Similarly to MSstatstTMT, the parameters μ, $\mathrm{Protei}n_{k}$, $\mathrm{Channe}l_{kc}$, and $\mathrm{Featur}e_{f}$ are unknown and of our primary interest. Note that the term $\mathrm{Channe}l_{kc}$ differs from the additive term $\mathrm{Channe}l_{c}$ in Equation (1). The additive term corresponds to the assumption that expression profiles of all proteins in a cluster follow the same pattern and differ only by a shift along the Y-axis. In contrast, $\mathrm{Channe}l_{kc}$ allows us to separately model each, possibly non-parallel, protein profile in the cluster. Parameters $\mathrm{Weigh}t_{fk}$ are unknown auxiliary parameters that describe the contribution of protein k to the abundance of feature f. In particular, setting all weights to $\frac{1}{\left|V(f)\right|}$ is equivalent to assigning each feature to every matching protein. Such weights can be used to aid protein inference based on the inclusion principle. We do not impose any distributional assumptions on $\varepsilon_{cf}$, making this approach adaptable to various types of noise. Similarly to Equation (2), protein-level summary for protein k=1,…,K in channel c=1,…,C is estimated as

(6) $${\hat{Y}}_{kc}=\hat{\mu }+{\hat{\mathrm{Protein}}}_{k}+{\hat{\mathrm{Channel}}}_{kc}$$

These summaries serve as input to statistical models that determine differential abundance of proteins. In this article, we use MSstatsTMT models defined in Equations (3) and (4).

### 2.2 Objective function for parameter estimation

We propose to fit the model separately for each protein cluster and each TMT mixture by minimizing

$$\begin{array}{l} \min\sum_{c=1}^{C}\sum_{f=1}^{F}L(X_{cf}-\mu -\mathrm{Featur}e_{f}-\\ \sum_{k\in V(f)}We\mathrm{igh}t_{fk}\left(\mathrm{Protei}n_{k}+\mathrm{Channe}l_{kc}\right)) \end{array}$$

where L is a loss function, and the optimization is done simultaneously with respect to μ, *Feature*, *Weight*, *Protein*, and *Channel*. The choice of loss function corresponds to different assumptions about the error term distribution $\varepsilon_{fc}$. For example, normal distribution of $\varepsilon_{fc}$ leads to $L_{2}(x)=x^{2}$, while Laplace distribution of $\varepsilon_{fc}$ leads to $L_{1}=|x|$. The latter leads to the procedure which is more robust towards the outliers, similar to the Tukey Median Polish approach in MSstats (Fink 1988). In practice, we observed that our algorithm with $L_{1}$ loss encounters the convergence issues, which may result from the loss non-differentiability. Therefore, our default implementation uses the smooth robust Huber loss (Huber 1992) given by

$$L_{H}(x,M)=\{\begin{array}{cc} 2M|x|-M^{2}, & |x|\geq M,\\ |x{|}^{2}, & |x|<M, \end{array}$$

where x is a scalar input and M is a positive hyperparameter tuned for each experiment. Low values of M ensure robustness of the estimates and convergence of the optimization procedure, while high values of M may make the estimates susceptible to outliers. Hence, Huber loss should be used with a small M parameter to ensure robustness. For example, case studies presented in this article used values of M=0.001 or $M=10^{-6}$. Supplementary Section S4.7 further discusses the importance of using a robust loss. The uncertainty of parameter estimates, in particular of the weights, is determined by the diversity of the quantitative profiles of the proteins in a cluster, as illustrated in Supplementary Section S4.1.

### 2.3 Optimization of the objective function

The model in Equation (3.1) is not linear in parameters $\mathrm{Weigh}t_{fk}$ and $\mathrm{Channe}l_{kc}$, as these parameters enter the loss function via the multiplicative terms $\mathrm{Weigh}t_{fk}C\mathrm{hanne}l_{kc}$. However, given fixed values of $\mathrm{Weigh}t_{fk}$, the model is linear in parameters $\mathrm{Channe}l_{kc}$. Similarly, given constant values of the remaining parameters, the model is linear in $\mathrm{Weigh}t_{fk}$. In both situations, all the loss functions in Section 3.2 are convex. A common practice for solving such biconvex problems is alternatively updating the two sets of parameters by fixing one of them and solving the convex optimization problem for the other. Therefore, we propose to estimate the parameters of the model with an iterative procedure outlined below, and described in more detail in Supplementary Section S2.1.

Following the alternate convex search approach (De Leeuw 1994), we initialize the optimization by estimating protein-level profiles based on unique features only. This allows us to fix the values of parameters *Protein* and *Channel*, and estimate the parameters *Weight.* Then we re-estimate *Protein* and *Channel* with the updated values of *Weight*. These two estimation steps are repeated until the difference between consecutive values of *Weight* is sufficiently small.

Unfortunately, the algorithm does not generally guarantee either local or global optimality of the solution (Gorski *et al.* 2007, Shen *et al.* 2017). However, in situations where each protein in a cluster has unique peptides, we observed no significant dependence of the solution on the starting point, and the algorithm usually achieved convergence to a meaningful solution after a small number of iterations.

### 2.4 Implementation

We implemented the proposed approach in a free and open source R package MSstatsWeightedSummary. The implementation takes as input a list of feature intensities identified and quantified by a spectral processing tool, in the same format as MSstatsTMT.

MSstatsWeightedSummary requires that each input peptide is annotated with all the proteins in a database that match its sequence. For data processing tools that do not provide that, MSstatsWeightedSummary includes a functionality that takes as input a user-specified database, and matches to each peptide all the proteins that contain its sequence. Moreover, MSstatsWeightedSummary offers functionalities for merging proteins identified by a same set of peptides, and for removing proteins identified by shared peptides only.

The package implements weighted summarization using Huber loss. The loss function is optimized with the CVXR R package for convex optimization (Fu *et al.* 2020). The missing feature intensities are ignored. For each protein cluster, the package outputs an object that extends a typical MSstatsTMT summary, and adds information about the estimated peptide–protein weights and the algorithm convergence. The package includes a functionality to combine MSstatsWeightedSummary output with MSstatsTMT summarization results before testing for differential abundance. More information is available in the package vignette.

## 3 Case studies

### 3.1 Overview

We evaluated the proposed approach in three case studies representing three types of experiments with TMT labeling: a protein degrader study with a group comparison design; thermal proteome profiling (TPP) with both repeated measures and group comparison designs; and relative PTM quantification in a group comparison design. Table 1 summarizes the case studies. Protein degrader and PTM case studies exhibited simpler structure of peptide sharing, as evident by the average number of proteins in a cluster only slightly larger than 1. Both parts of the TPP case study exhibited more complex structure. Hence, unlike in case of other datasets, subset proteins were retained and provided summaries include proteins that were only identified by shared peptides. Removing such proteins would lead to a loss of one-third of all peptide ions. In clusters that consist of only shared peptides, selecting a leading protein may not be obvious.

**Table 1.** Including peptides matching to multiple proteins changed the number of quantifiable proteins in each case study.^a^

		Case study			
		1	2a	2b	3
Number of protein labels	Original	7482	7043	8447	26 004
	Proposed	6323	11 084	25 043	24 809
Number of peptide ions	Original	81 851	89 423	164 863	43 585
	Proposed	73 881	90 223	165 906	43 585
Number of protein clusters	Proposed	5818	5699	6559	22 285
Mean number of proteins per cluster	Proposed	1.09	1.94	3.81	1.11
Mean number of shared peptides per cluster	Proposed	7.06	15.2	24.8	1.71

a Lines 3 and 4 count both trivial clusters consisting of single proteins and their unique peptides, and non-trivial clusters. Line 5 describes non-trivial clusters only. Case study 2a refers to the OnePot portion of the study, while 2b refers to TPP part. The large difference in number of protein labels between original and proposed processing in the latter was due to use of the *Master Protein Accessions* column of the Proteome Discoverer output which by design groups multiple proteins under a single label.

Overall, the experiments also included protein clusters of different sizes, with varying amounts of unique and shared information. The clusters affected the number of quantifiable proteins, and served as the basis for the case studies. We also evaluated the proposed approach in computer simulations.

### 3.2 Case study 1: protein degrader

#### 3.2.1 Experimental design

This previously unpublished case study evaluated BET bromodomain degradation by GNE-0011 BET binder in EOL-1 cells. Samples treated with either DMSO (control group) or GNE-001 (treatment) were measured at 0, 30, 60, 120, and 480 min in a group comparison design to estimate changes on protein abundances in time. The experiment only included one biological replicate per time and condition. The samples were labeled with TMT-10plex in a single TMT mixture. Supplementary Section S3 provides additional details.

#### 3.2.2 Data acquisition and processing

Mass spectra were acquired on an Orbitrap Fusion Lumos Mass Spectrometer (ThermoFisher Scientific) coupled to an RSLCnano U3000 liquid chromatography system (ThermoFisher Scientific), and are available in MassIVE MSV000094252, password BRD4Degrader. The spectra were searched against Swissprot human protein database (version 2017.08) and processed with in-house software and the Mojave algorithm (Zhuang *et al.* 2013). The original processing matched shared peptides to an arbitrary selected single protein. As part of the MSstatsWeightedSummary pre-processing, we matched each peptide to all the proteins that contain its sequence, and removed proteins identified by shared peptides only.

#### 3.2.3 Protein cluster

**Figure 2.**
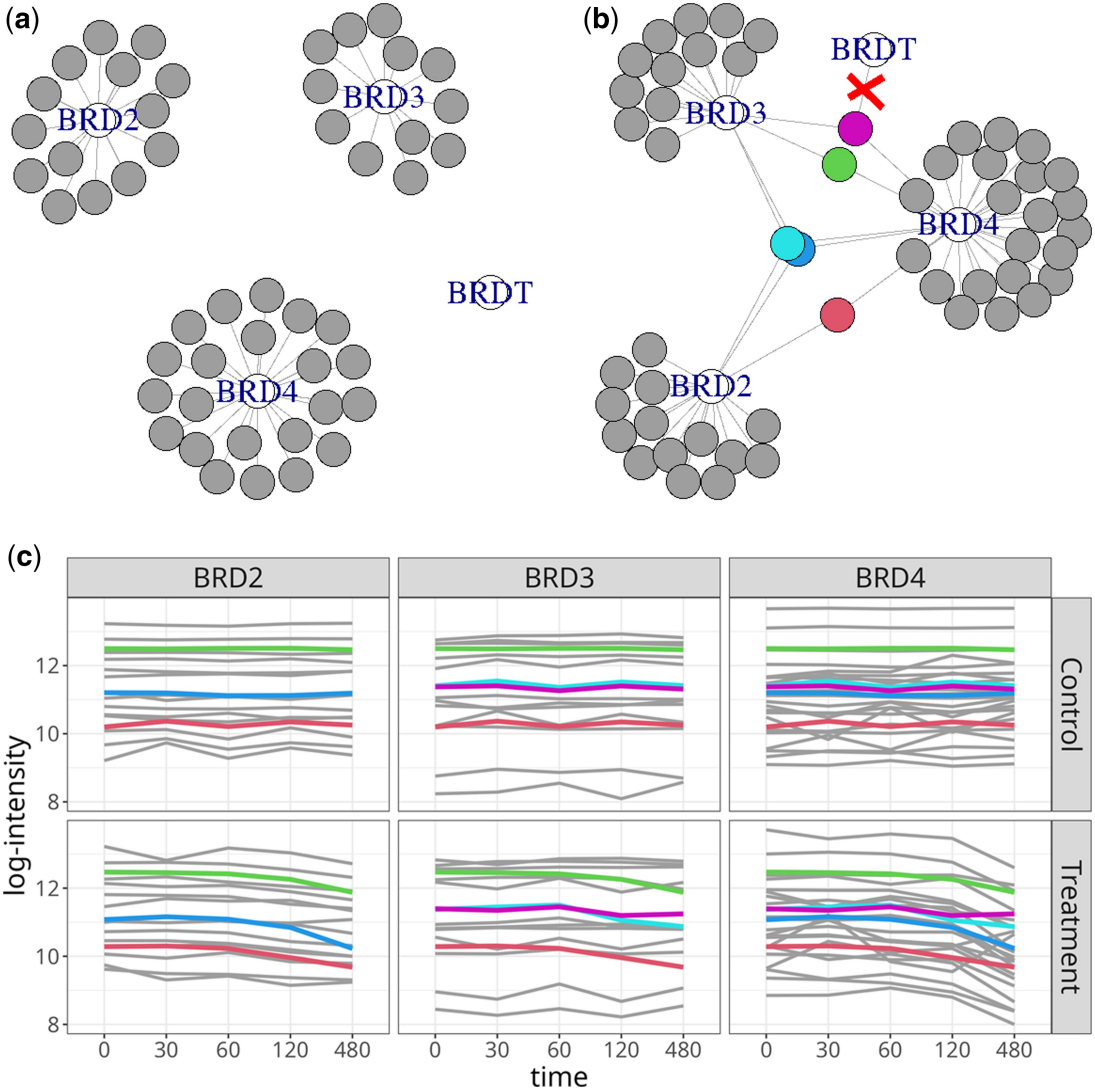
Protein degrader: proteins RD2, BRD3, and BRD4. Modeling the contribution of shared peptides transformed the disjoint sub-graphs into a connected graph with heterogeneous peptide patterns. (a) Proteins characterized by unique peptides. Grey nodes: unique peptides. Edges: matches between peptide and protein sequence. (b) As in (a), but with shared peptides (colored nodes). Protein BRDT did not have unique peptides and was filtered out by the proposed processing. (c) Quantitative profiles of the peptides. Line colors match node colors in (a) and (b). At later time points, the unique quantitative profiles of the three proteins diverged. The patterns of shared peptides deviated from the patterns of the unique peptides.

We considered an example cluster of four proteins BRDT, BRD2, BRD3, and BRD4 with sequence similarity of approximately 60% (Madeira *et al.* 2022). The peptide–protein graph for this cluster along with a profile plot is shown in Fig. 2. The cluster contained five shared peptides, of which five matched to BRD4, four matched to BRD3, and three matched to BRD2. This investigation had no ground truth of differential abundance. However, a western blot assay confirmed that both BRD2 and BRD4 had significant BET bromodomain degradation, but with different rates.

### 3.3 Case study 2: thermal profiling

#### 3.3.1 Experimental design

Xu *et al.* (2021) investigated protein targets in K562 cell lysate treated with Staurosporine (kinase inhibitor) as compared to treatment with a control DMSO. The authors performed two thermal profiling experiments that studied drug target engagement: TPP and its OnePot counterpart.

The TPP experiment treated the samples with Staurosporine at 25× the concentration of DMSO. It utilized a repeated measures design, whereby each biological sample was heated at 11 increasingly high temperatures. Two biological samples per condition, heated at different temperatures, were labeled with TMT-10plex, and each allocated to a different TMT mixture. All the proteins were expected to decrease in abundance in response to the treatment, but at a different rate. Here, we compared protein abundances of treated versus control samples at mid-temperature point.

The OnePot experiment involved physical pooling of all of the temperature-subjected aliquots of a same biological replicate prior to isobaric labeling. Therefore, this version of the experiment had a group comparison design. The OnePot experiment included not one but four concentrations of Staurosporine (1×,5×,10×,25×) and DMSO (control), and three biological replicates per condition. The pooled samples were labeled with the first 15 labels from TMTpro 16plex in a single mixture. Here, we also compared protein abundances of samples treated with Staurosporine at 25× the concentration of DMSO to the controls. Since the OnePot experiment had a larger number of concentrations and of biological replicates it was expected to produce more accurate conclusions.

#### 3.3.2 Data acquisition and processing

In both experiments, mass spectra were acquired with an Orbitrap Fusion Lumos Tribrid mass spectrometer (Thermo Fisher Scientific), searched against Homo Sapiens Swissprot database (v2017-10–25) and processed using Proteome Discoverer 2.4 (Orsburn 2021), and made available online (links can be found in the README file of a GitHub repository linked above). The original processing used the protein inference algorithm by Proteome Discoverer. For each shared peptide, the algorithm concatenated the identifiers of all the matching proteins that had at least one unique peptide. As part of the MSstatsWeightedSummary pre-processing, we matched the identified peptides to all the proteins in the database, merged proteins with identical sets of peptides, and removed proteins identified by a single shared peptide. In the TPP experiment, the lowest temperature was used as between-mixture normalization channel, and the highest temperature was discarded from the analysis. Details of data acquisition and processing can be found in the original article.

#### 3.3.3 Protein clusters

Although the experiment had no direct ground truth, we used a set of known interactors (Figueroa-Navedo 2023) from the KinHub database (Davis *et al.* 2011, Eid *et al.* 2017) as a proxy of true changes in protein abundance. Moreover, we compared the detected changes in abundance to those of the more sensitive OnePot portion of the study.

We used non-trivial clusters of proteins that included at least one known interactor and considered proteins with at least one unique peptide. In the TPP part of the study, there were 27 such clusters which consisted of 75 proteins. In the OnePot portion of the study, there were 34 such clusters and 93 proteins. For each non-trivial cluster, we compared the outcome of tests for differential abundance with the proposed summarization to the results based on summarization with unique peptides or all peptides matching to each protein.

Moreover, we considered a cluster of three proteins P16591, P16591-2, and P16591-3. Proteins P16591 and P16591-2 were identified by three peptides unique to these two proteins, and these two proteins could not be distinguished. Therefore, the proposed processing merged them into a single protein identifier. Proteoform P16591-3 was approximately 90% similar to the first two proteins (Madeira *et al.* 2022) but was identified by two unique peptides. This protein was present in both the set of known interactors and in the list of differentially abundant OnePot proteins based on the original data processing.

### 3.4 Case study 3: multi-site PTM

#### 3.4.1 Experimental design

Maculins *et al.* (2021) quantified the abundance of total protein and phosphorylation in wildtype (WT) and ATG16L1-deficient (cKO) samples of primary murine macrophages uninfected and infected with *Shigella flexneri*. Quantification was performed at three time points: uninfected, early infection, and late infection, in a group comparison design. This study made nine comparisons: KO Early-WT Early, KO Late-WT Late, KO Uninfected-WT Uninfected, KO Early-KO Uninfected, KO Late-KO Uninfected, WT Early-WT Uninfected, WT Late-WT Uninfected, Infected-Uninfected, and KO-WT, while adjusting changes of modified peptides for changes in global protein abundance. Twenty-two biological samples were split between two 11-plex TMT mixtures. Mixture 1 had one replicate of uninfected WT and two replicates of uninfected cKO. Mixture 2 had one replicate to uninfected cKO and two to uninfected WT.

#### 3.4.2 Data acquisition and processing

Mass spectra were acquired on an Orbitrap Fusion Lumos mass spectrometer coupled to an EASY nanoLC-1000 (or nanoLC-1200) (ThermoFisher) liquid chromatography system. Spectra were searched against a UniProt mouse and *S. flexneri* protein sequences database and processed with the Mojave algorithm (Zhuang *et al.* 2013). Modifications searched included phosphorylation on serine, threonine, and tyrosine. Localization was performed with a modification of AScore algorithm (Beausoleil *et al.* 2006). The dataset is available in MassIVE under identifier MSV000085565. In the original processing, peptides with multiple modifications were assigned a new modification that concatenated all the sites. As part of the MSstatsWeightedSummary pre-processing, we modified the original site annotation by assigning peptides with multiple modification sites to all possible sites. Since the experiment lacked a normalization channel, all the analyses proceeded without normalization. Details of data acquisition and processing can be found in the original article.

#### 3.4.3 Protein cluster

We considered an example of two modification sites S236 and S240 on a single protein E9Q6J5. Since this experiment had no ground truth of differential abundance, we compared the precision of the proposed approach in terms of the characterized modification sites to previously published results (Kohler *et al.* 2023b) available in a MassIVE.quant repository RMSV000000357.

### 3.5 Simulated data and resampled data

To evaluate the proposed approach in a setting with known ground truth, we conducted extensive computer simulations. We simulated a cluster of five proteins, and simulated peptides such that each pair of proteins shared peptides. We simulated the peptide-level abundances according to Equation (3.1), and protein-level abundances according to the MSstatsTMT model in Equation (3). We varied effect sizes, numbers of shared and unique peptides, and number of biological replicates. We also conducted a resampling study based on Case study 1. Since the BRD cluster had more than 10 unique peptides per protein, we randomly sampled their subsets in various configurations. For each configuration, we created protein-level summaries, performed group comparisons, and compared the results to those obtained with all the available unique peptides. We evaluated the performance of the proposed approach in terms of the mean-squared error (MSE) of ${\;\text{log}\;}_{2}$-fold change estimation with respect to the ground truth (for the simulation), or with respect to the results obtained with all the available unique peptides (for the resampling study based on Case study 1). Supplementary Sections S3.1 and S3.2 provide details of both model- and resampling-based simulations, and define the evaluation metrics.

## 4 Results

Since most existing methods for relative protein quantification with shared peptides (Section 1.1.2) are not directly applicable to experiments with TMT labels, and are incompatible with experiments with complex designs such as repeated measures, we compared the proposed approach to an analysis that only uses unique peptides (which we also refer to as unique-only approach), and to naïve inclusion (i.e. an analysis that uses all the available peptides for all the proteins in a cluster as if they were unique, also referred to as all-peptides approach). To enable the evaluation, we focused on clusters where each protein is identified by at least one unique peptide in addition to shared peptides. Supplementary Sections S4.5 and S4.6 discuss the properties of the proposed approach in situations where some proteins lack unique peptides from the perspective of convergence and quantification of subset proteins, respectively. The proposed approach was fitted using version 0.99.6 of the MSstatsWeightedSummary package. For each case study, the loss function used Huber norm. Case studies 1 and 3 used a value of $M=10^{-3}$, while case study 2 used a smaller value of $M=10^{-6}$. The estimation was done by ignoring missing values while fitting the proposed model. Supplementary Section S4.2 studies the impact of modeling shared peptides while varying the extent of unique peptides and with a more complex peptide–protein structure.

### 4.1 Accounting for shared peptides produced a more parsimonious set of testable proteins

#### 4.1.1 Protein degrader

While the original processing assigned each shared peptide to an arbitrary protein, the proposed approach modeled the weighted contribution of each shared peptide to all the possible proteins. This, combined with removing proteins identified only by shared peptides, resulted in an overall reduction of the number of testable proteins (Table 1). Moreover, it enabled proper modeling of the peptide–protein structure. This is illustrated in the case of BRD cluster in Fig. 2a and b. The BRDT protein was only identified by a single shared peptide. It was assigned to BRD3 by the original processing but was removed from the analysis by the proposed approach.

#### 4.1.2 Thermal profiling, OnePot

Similarly, Table 1 shows an overall decrease in the number of protein labels after the proposed processing. Table 2 illustrates this in the selected protein cluster. The original Proteome Discoverer processing combined peptides from three proteins Q7Z5L9, Q7Z5L9-2, and Q9H1B7 into five protein groups. Groups labeled with multiple proteins consisted of peptides that match to more than one protein. Simplified protein set is beneficial from the perspective of downstream statistical analysis which uses multiple testing correction.

**Table 2.** Thermal profiling, OnePot: inclusion of shared peptides simplified the set of testable proteins.^a^

	Proteome Discoverer protein groups				
Proposed				Q7Z5L9;	Q7Z5L9;
protein	Q7Z5L9	Q7Z5L9-2	Q9H1B7	Q7Z5L9-2	Q7Z5L9-2;
group					Q9H1B7
Q7Z5L9	1	0	0	15	3
Q7Z5L9-2	0	2	0		
Q9H1B7	0	0	13	0	

a The Proteome Discoverer protein inference algorithm allocated peptides from proteins Q7Z5L9, Q7Z5L9-2, and Q9H1B7 into five distinct protein groups, namely Q7Z5L9, Q7Z5L9-2, Q9H1B7, Q7Z5L9 and Q7Z5L9-2, Q7Z5L9; Q7Z5L9-2; and Q9H1B7. In contrast, the proposed approach did not expand beyond the three protein labels. The table counts the number of peptides in each allocation. For example, while Proteome Discover allocated 15 peptides to a new protein group Q7Z5L9; Q7Z5L9-2, the proposed approach distributed each of the 15 peptides between the existing proteins Q7Z5L9 and Q7Z5L9-2 with contribution weights based on the quantitative profiles.

#### 4.1.3 Multi-site PTM

In the approach proposed by Kohler *et al.* (2023b), peptides with multiple modification sites were viewed as carrying a separate concatenated multi-site modification. Across the entire data set, modeling the contributions of peptides covering multiple sites instead of creating a new multi-site modification, combined with filtering, reduced the number of testable sites. In the example cluster (Table 3), the original processing assigned two peptides for each of S236, S240, and concatenated S236_S240, and the proposed approach only kept the individual sites.

**Table 3.** Multi-site PTM: inclusion of peptides covering multiple modification sites simplified the set of testable sites.^a^

Proposed PTM sites of protein E9Q6J	Original PTM sites of protein E9Q6J		
	S236	S240	S236_S240
S236	2	0	2
S240	0	2	

a For peptides of protein E9Q6J covering sites S236 and S240, the original processing (Kohler *et al.* 2023b) created a new multi-site modification S236_S240. In contrast, the proposed approach did not expand beyond the two modification sites. The table counts the number of peptides in each allocation. While the original processing allocated two peptides to the new S236_S240, the proposed approach distributed each of the two peptides between the existing S236 and S240, with contribution weights based on the quantitative profiles.

### 4.2 Shared peptides improved ${\;\text{log}\;}_{2}$-fold change estimation for proteins with few unique peptides

#### 4.2.1 Protein degrader

**Figure 3.**
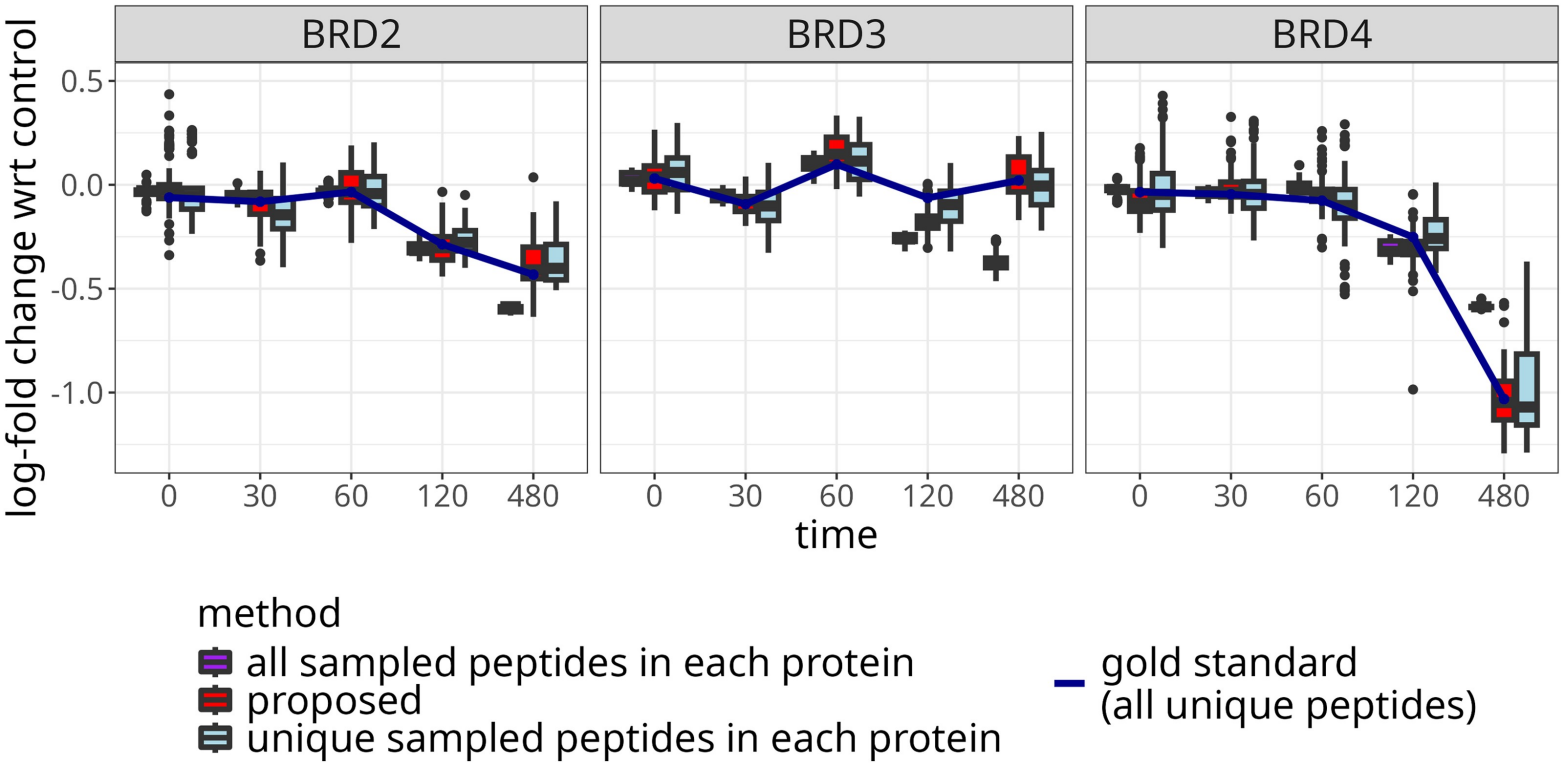
Protein degrader: modeling the contributions of shared peptides improved the ${\;\text{log}\;}_{2}$-fold change estimation for the BRD cluster. The boxplots summarize 100 instances of randomly selecting two unique peptides per protein and all five shared peptides, and estimating ${\;\text{log}\;}_{2}$-fold changes with respect to the control sample at the same time point. The solid line denotes ${\;\text{log}\;}_{2}$-fold changes estimated using all the available unique peptides, i.e. the ground truth. Narrower boxes with median closer to the blue line indicate better performance. The change in protein abundance for BRD2 and BRD4 was confirmed experimentally by Western blot.

Since the study had no known ground truth, we investigated the benefits of modeling the contributions of shared peptides by peptide resampling. Figure 3 summarizes the estimated ${\;\text{log}\;}_{2}$-fold changes in 100 instances of randomly selecting two unique peptides per protein and all five shared peptides. The proposed approach reduced the bias as compared to the estimation with all the peptides, and reduced the variance as compared to the estimation with a subset of the unique peptides.

**Figure 4.**
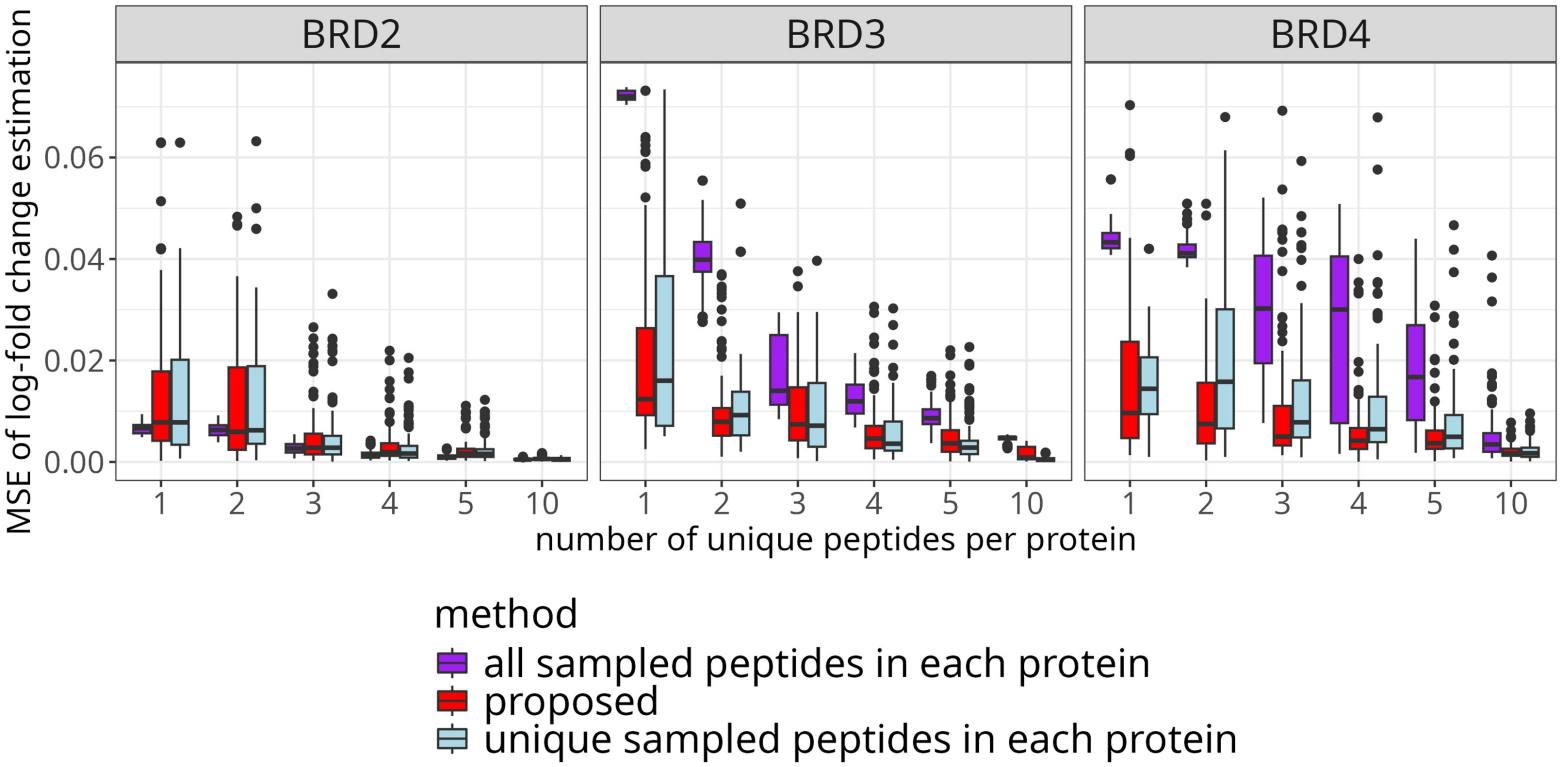
Protein degrader: modeling the contributions of shared peptides improved the mean-squared error of ${\;\text{log}\;}_{2}$-fold change estimation for the BRD cluster, particularly with few available unique peptides. The boxplots summarize 100 instances of randomly selecting unique peptides per protein and all five shared peptides, and estimating ${\;\text{log}\;}_{2}$-fold changes with respect to the control sample at the same time point, where the ${\;\text{log}\;}_{2}$-fold changes calculated based on all available unique peptides served as ground truth. The MSE was plotted as function of the number of unique peptides per protein. Lower and narrower boxes indicate better performance. While all-peptides achieved lower error for the BRD2 protein, it overfitted to that particular quantitative pattern, resulting in much higher errors for the other proteins.

Figure 4 details the results of the same resampling-based investigation in terms of mean-squared error, as function of the number of unique peptides per protein. The proposed approach improved the accuracy of the estimation as compared to using all the peptides, or using a selected subsets of the unique peptides, and was particularly effective when the number of unique peptides per protein was small.

#### 4.2.2 Multi-site PTM

**Figure 5.**
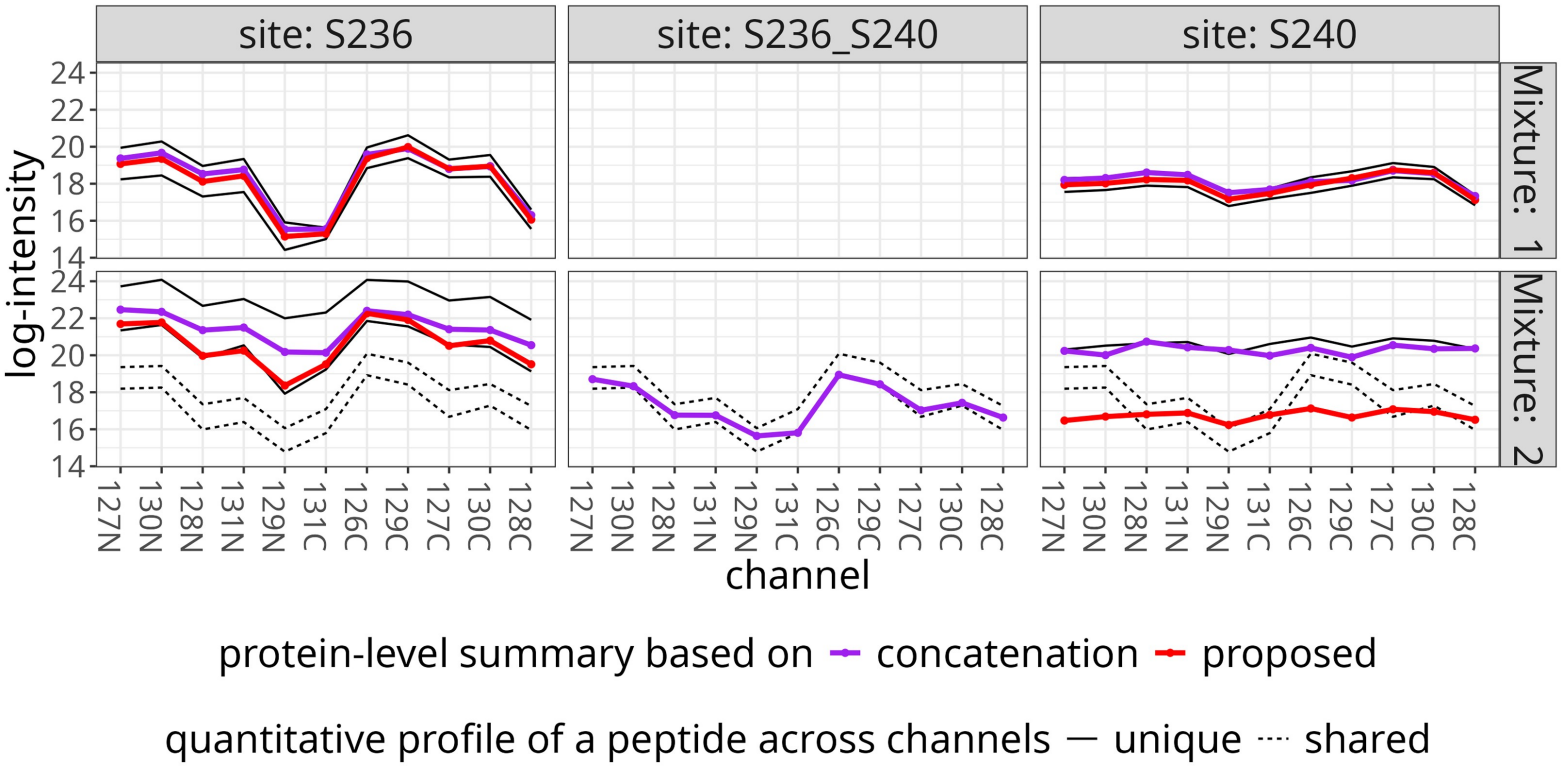
Multi-site PTM: modeling the contributions of shared peptides improved the estimation of site-specific quantitative profiles of protein E9Q6J. Mixture 2 contained two peptides modified at sites S236 and S240. The proposed approach allocated each peptide to site S236 with weight 1. It eliminated the concatenated modification without distorting the quantitative patterns in the summary for site S240 (right panel), while the pattern for site S236 (left panel) better captures the pattern observed in the other mixture.

Figure 5 visualizes the improved ${\;\text{log}\;}_{2}$-fold change estimation in the selected cluster. Mixture 2 contained two peptides modified both at site S236 and site S240, however their quantitative profiles resembled closely the peptides with S236 alone. The proposed approach allocated these peptides to S236, each with weight 1. This reduced the total number of modification sites as compared to the concatenation approach. It improved the similarity of estimated quantitative profiles in Mixtures 1 and 2 as compared to the estimation using all the peptides for all the sites. Supplementary Section S4.3 shows that in this example the estimated ${\;\text{log}\;}_{2}$-fold changes tended to be larger in absolute value.

#### 4.2.3 Model-based simulation

**Figure 6.**
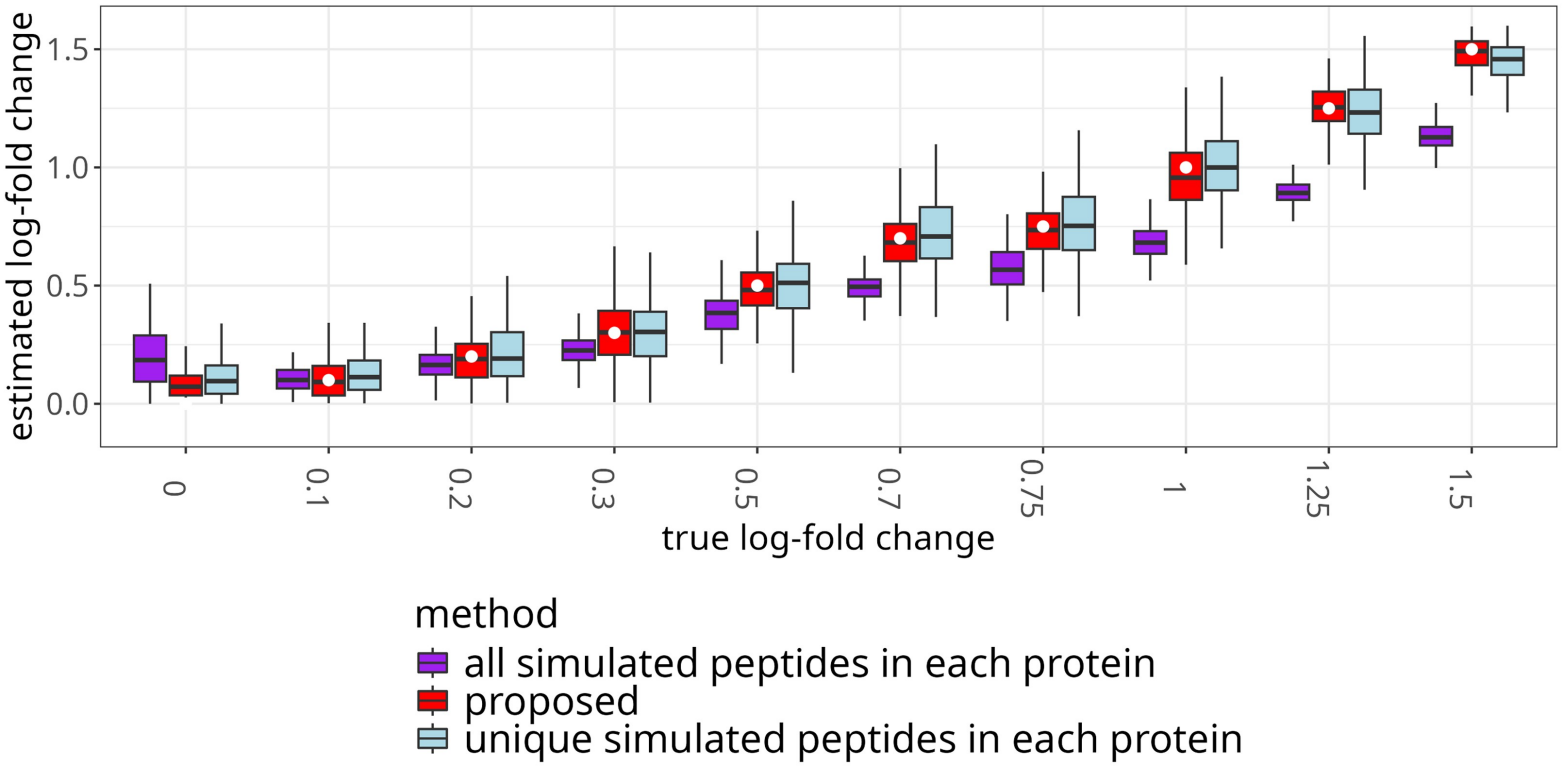
Simulated data: modeling the contribution of shared peptides improved the estimation of a range of ${\;\text{log}\;}_{2}$-fold changes. White dots indicate true values. The boxplots summarize 50 instances of simulating five proteins with two biological replicates, two unique peptides per protein, normal random error with a standard deviation of 0.2, with a range of true ${\;\text{log}\;}_{2}$-fold changes. Narrower boxes with median closer to the white dot indicate better performance.

In order to investigate the estimation of ${\;\text{log}\;}_{2}$-fold change in a context of known ground truth, we simulated 50 instances of five proteins with two biological replicates, two unique peptides per protein, and five shared peptides per pair of proteins, with a range of true ${\;\text{log}\;}_{2}$-fold changes. The simulation (Fig. 6) leads to the same conclusions as the experimental datasets. The proposed approach reduced the bias of the estimation as compared to using all the peptides, and reduced the variance of the estimation as compared to using the unique peptides only. The difference was particularly pronounced for large absolute values of ${\;\text{log}\;}_{2}$-fold change.

### 4.3 Shared peptides improved robustness of protein summarization for proteins with noisy unique peptides

#### 4.3.1 Protein degrader

**Figure 7.**
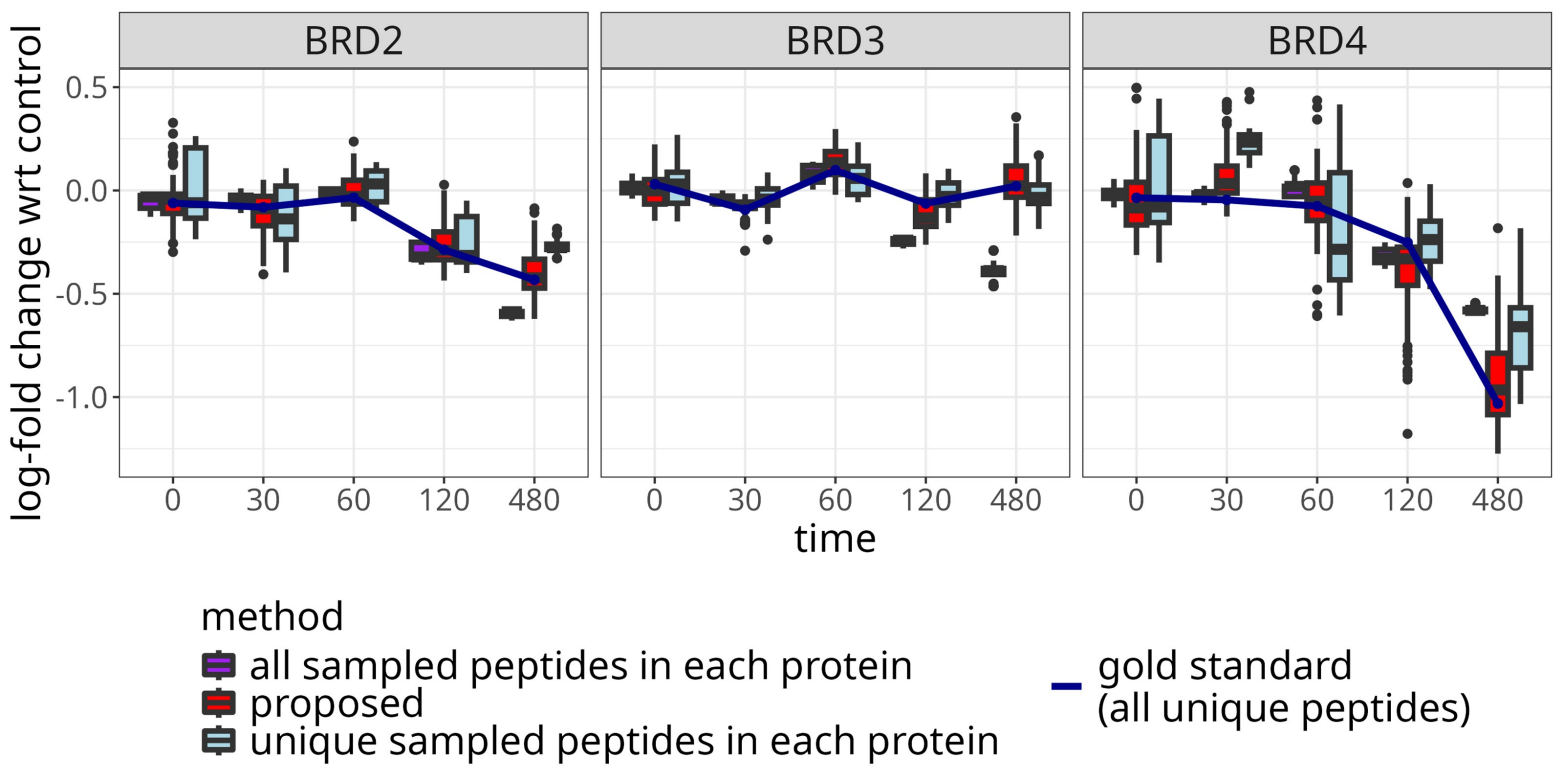
Protein degrader: modeling the contribution of shared peptides improved ${\;\text{log}\;}_{2}$-fold change estimation for the BRD cluster in presence of noisy unique peptides. The boxplots summarize 100 instances of randomly selecting two unique peptides per protein and all five shared peptides, and estimating ${\;\text{log}\;}_{2}$-fold changes with respect to the control sample at the same time point. One unique peptide per protein was noisy. The solid line denotes ${\;\text{log}\;}_{2}$-fold changes estimated using all the available unique peptides, i.e. the ground truth. Narrower boxes with median closer to the blue line indicate better performance. The change in protein abundance for BRD2 and BRD4 was confirmed experimentally by Western blot.

Interferences and measurement errors may produce noisy peptides, i.e. peptides with irregular quantitative patterns that differ from the majority of other peptides of a same protein. We once again used peptide resampling to evaluate the impact of the presence of unique but noisy peptides on the ${\;\text{log}\;}_{2}$-fold change estimation. In each resampling instance, we sampled a fixed number of unique peptides and used all the available shared peptides. The unique peptides included a single noisy peptide for each protein, selected from the pool of three unique peptides with the lowest average correlation to the other peptides matching the same protein. The proposed approach reduced the bias of ${\;\text{log}\;}_{2}$-fold change estimation as compared to using all the peptides or selected unique peptides (Fig. 7). In particular for BRD4, noisy unique peptides unduly influenced protein-level summaries based on unique peptides, while using all the peptides underestimated the change in abundance.

### 4.4 Modeling the contribution of shared peptides balanced the sensitivity and the specificity of detecting differentially abundant proteins

#### 4.4.1 Computer simulations

We evaluated the ability of the proposed approach to distinguish differentially abundant proteins for a range of ${\;\text{log}\;}_{2}$-fold changes by computer simulation as described in Supplementary Section S3.2. The simulation generated a cluster of five proteins, where three proteins were differentially abundant, and each pair of proteins shared peptides. We tested the proteins for differential abundance in a design that mimics the protein degrader case study with five conditions. However, for simplicity, the comparisons were made between a single reference group and each of the remaining four conditions. The MSstatsTMT modeling approach was used, and the *P*-value cutoff was set to 0.05. The *P*-values were not adjusted for multiple testing to avoid the dependence of the conclusions on different total numbers of tests between the evaluations.

**Figure 8.**
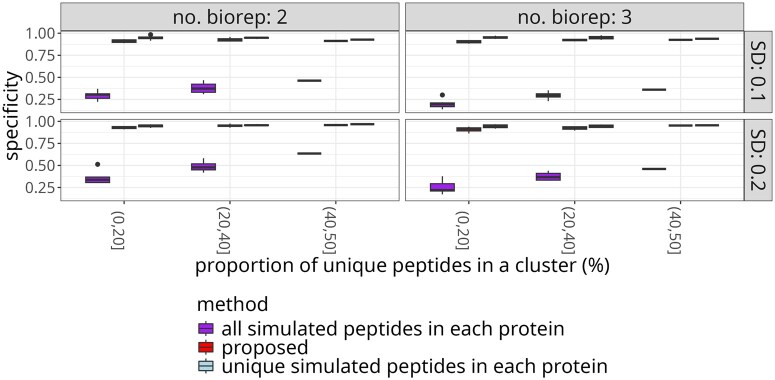
Simulated data: the proposed approach increased the specificity of detecting true differentially abundant proteins as compared to the analysis using all the peptides. Five proteins with three differentially abundant ones and a range of true ${\;\text{log}\;}_{2}$-fold changes were simulated. *Y*-axis: specificity of the test for differential abundance. The panels distinguish two versus three biological replicates per condition, as well as the standard deviations of the random error. Narrower and higher boxes with median closer to 1 indicate better performance.

Figure 8 summarizes the specificity of differentially abundant proteins across 50 replicates of the experiment. The proposed approach increased the specificity of the results as compared to the all-peptides approach. The all-peptides approach overfitted to peptides with the largest differences between conditions, and produced many false positive detections. Thus, this approach is not suitable for detection of differentially abundant proteins in clusters that include proteins that do not change between conditions.

**Figure 9.**
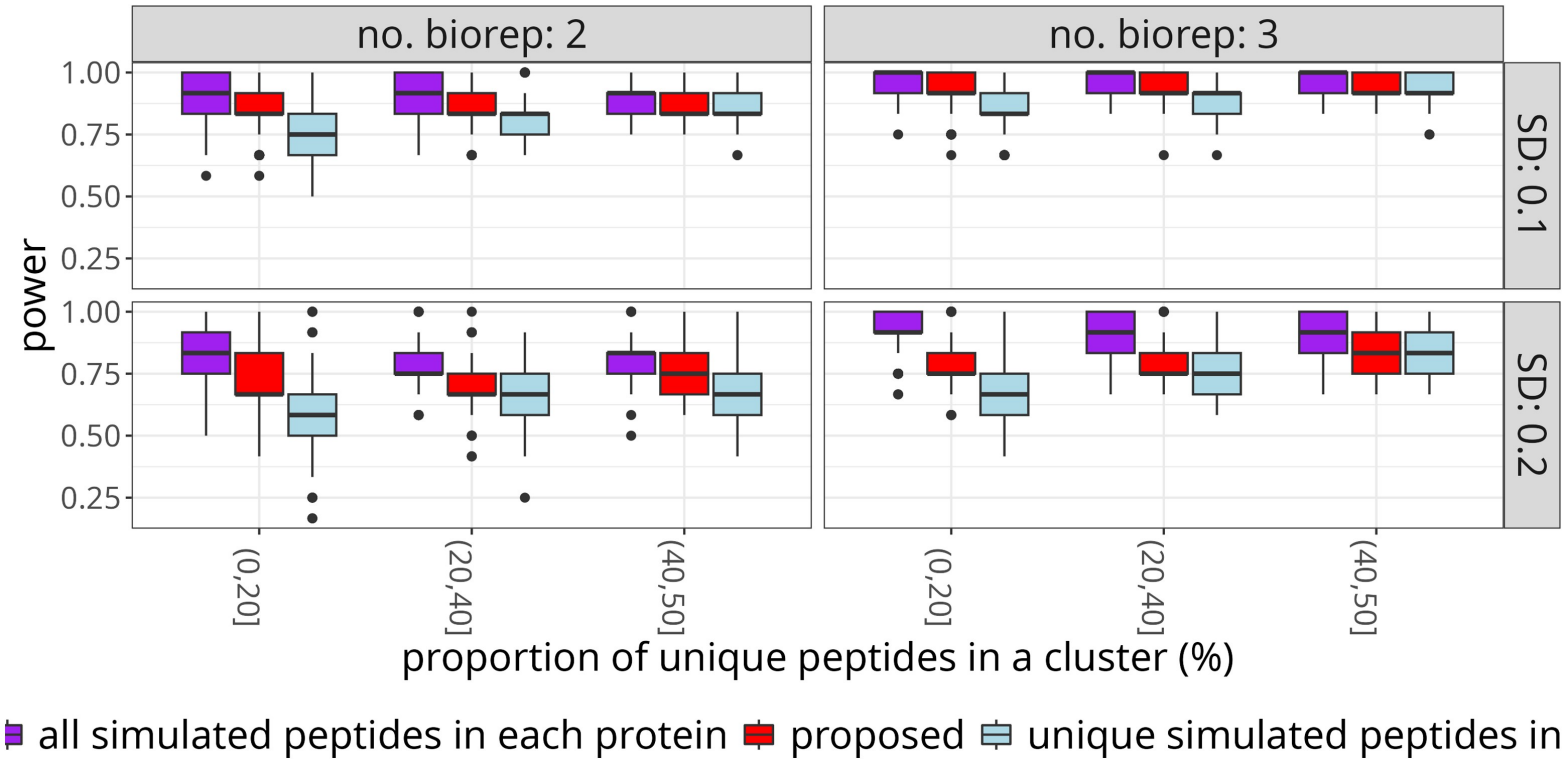
Simulated data: the proposed approach increased the sensitivity of detecting differentially abundant proteins as compared to the analysis using unique peptides only. The boxplots summarize 50 instances of the simulation of five proteins with three differentially abundant ones and a range of ${\;\text{log}\;}_{2}$-fold changes. *X*-axis: true ${\;\text{log}\;}_{2}$ fold change. *Y*-axis: sensitivity of the test for differential abundance. The panels distinguish two versus three biological replicates per condition and sizes of the standard deviation of the random error term. Narrower boxes with median closer to 1 indicate better performance.

Figure 9 summarizes the sensitivity of the proposed approach over 50 instances of the simulation, as a function of the true ${\;\text{log}\;}_{2}$-fold change. The proposed approach increased the sensitivity as compared to the analysis using unique peptides only. With the increased sample size due to the inclusion of shared peptides, proposed approach produced smaller standard errors compared to the unique-only approach. While all-peptides approach appears to perform well, its high sensitivity is associated with very large error rates, as seen by the low specificity.

#### 4.4.2 Thermal proteome profiling

**Figure 10.**
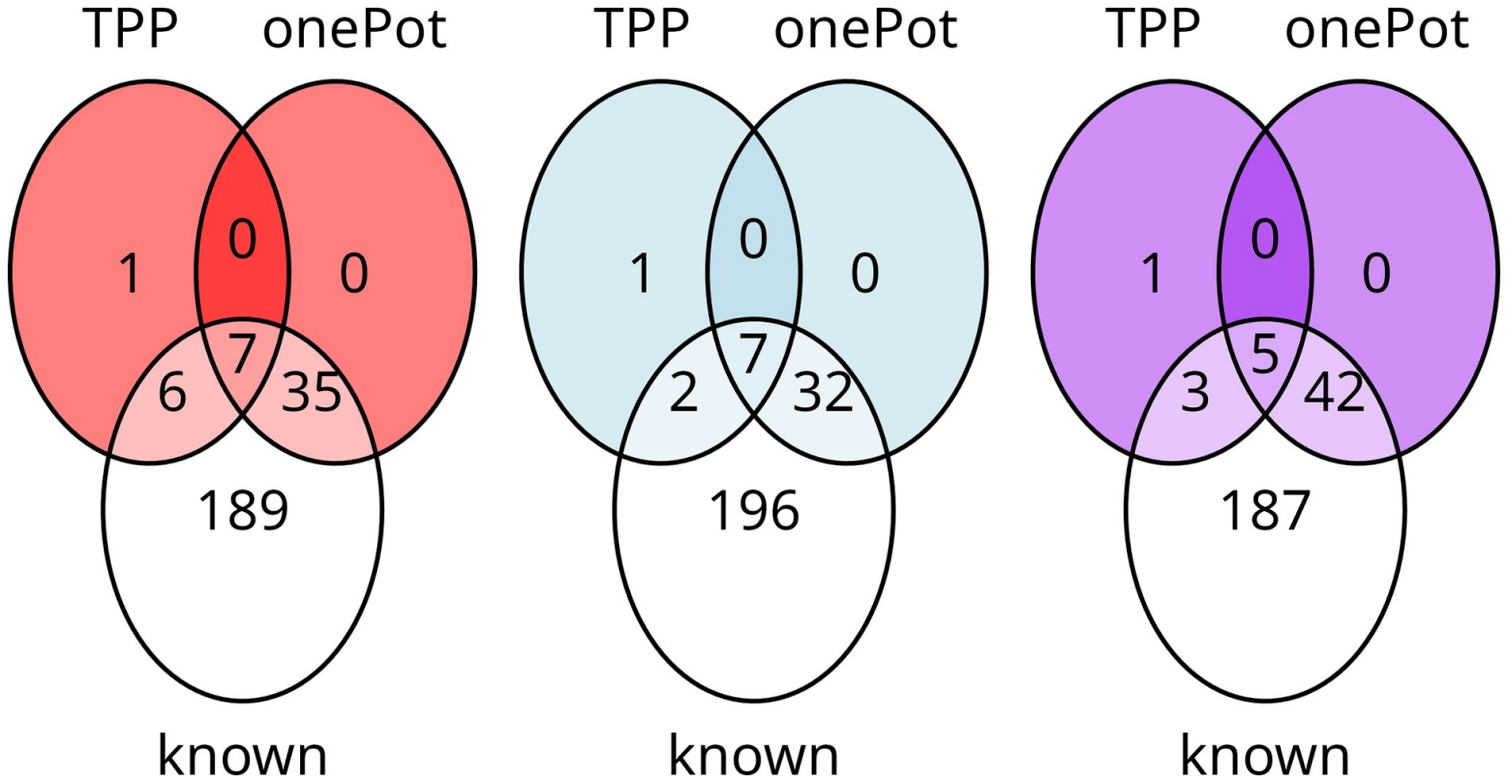
Thermal proteome profiling: the proposed approach improved sensitivity of differential abundance testing compared to the unique-only approach. Left-hand side plot describes the proposed summarization approach, while center and right-hand side plots describe unique-only and all-peptides approaches, respectively. In the TPP portion of the study, proposed approach achieved the highest sensitivity. In the OnePot portion it improved on the sensitivity of unique-only analysis. As indicated by the simulation study, high sensitivity of the all-peptides approach typically came at a cost of low specificity.

We evaluated the ability of the proposed approach and summarization based on either unique peptides or all matching peptides to capture the differential abundance. Figure 10 compares the three approaches to summarization (proposed weighted summarization, unique-only analysis, and all-peptides approach) from this perspective by showing the overlap in discovered differentially abundant proteins among the known interactors between the three approaches (proposed weighted summarization, unique-only analysis, and all-peptides approach).

Supplementary Section S4.4 details the comparison between protein-level summaries for one cluster of proteins for which inclusion of shared peptides helped discovering a differentially abundant known protein interactor in the TPP portion of this study, and which was not identified as differentially abundant by alternative approaches.

## 5 Discussion

We introduced an approach for modeling the contributions of peptides shared across protein clusters to individual quantitative protein-level summaries. The proposed approach is most effective when the proteins in a cluster have a limited number of unique peptides, and each unique protein has a distinct quantitative profile. Although the number of non-trivial protein clusters with such properties is typically small, our results indicate that the modeling can substantially impact the biological conclusions for some proteins. The impact stems from a more accurate estimation of ${\;\text{log}\;}_{2}$-fold changes and their standard errors, as well as from an overall reduced number of testable proteins and alleviated multiple testing.

The proposed approach is currently implemented in the context of experiments with TMT labeling. Although it is conceptually applicable to label-free experiments, it must be adapted to a larger extent of missing values in label-free measurements. In the presence of shared peptides, treatment of missing values such as imputation must be considered for all the proteins in a cluster jointly, and requires multivariate assumptions that differ from those implemented by most standard approaches. Such extensions will be the focus of our future work. Despite the opportunities for future extensions, we believe that the proposed approach in its current form is already valuable for many investigations.

## 6 Conclusion

We introduced a statistical approach to the problem of joint estimationof abundance profiles across biological samples for proteins or post-translational modifications that share peptides. The proposed modelenabled a more precise estimation of changes between conditions insuch cases. The method was implemented in a free and open source Rpackage MSstatsWeightedSummary compatible with the MSstatsworkflow.

## Supplementary Material

btaf046_Supplementary_Data

## Data Availability

The data underlying this article can be accessed via the GitHub repository doi:10.5281/zenodo.14656053.
