# Supplementary material for: Relative quantification of proteins and post-translational modifications in proteomic experiments with shared peptides: a weight-based approach
Source: Bioinformatics. 2025 Jan 31;41(3):btaf046. doi: 10.1093/bioinformatics/btaf046 (PMC11879648; doi:10.1093/bioinformatics/btaf046)
Supplement: btaf046_Supplementary_Data [file btaf046_supplementary_data.zip › MSstatsWeightedSummary__relative_protein_quantification_with_shared_peptides-SI-final.pdf]

Relative quantification of proteins and post-translational modifications in proteomic experiments with shared peptides: a weight-based approach

Supplementary materials

Mateusz Staniak[1,2], Ting Huang[3,4], Amanda M. Figueroa-Navedo [4], Devon Kohler[3,4], Meena Choi[5], Trent Hinkle[5], Tracy Kleinheinz[6], Robert Blake[6], Christopher M. Rose[5], Yingrong Xu[7], Pierre M. Jean Beltran[8], Liang Xue[8], Małgorzata Bogdan[1,9], Olga Vitek[3,4,*]

[1]Faculty of Mathematics and Computer Science , University of Wrocław, Wrocław, Poland
[2]Centre for Statistics, Hasselt University, Hasselt, Belgium
[3]Khoury College of Computer Sciences, Northeastern University, Boston, MA, US
[4]Barnett Institute of Chemical and Biological Analysis, Northeastern University, Boston, MA, US
[5]Department of Microchemistry, Proteomics, and Lipidomics, Genentech, Inc. South San Francisco, CA, US
[6]Department of Biochemical and Cellular Pharmacology, Genentech, Inc. South San Francisco, CA, US
[7]Discovery Sciences, Pfizer Inc., Groton, CT, US
[8]Machine Learning and Computational Sciences, Pfizer Inc., Cambridge, MA, US
[9]Department of Statistics, Lund University, Lund, Sweden

[*]To whom correspondence should be addressed: o.vitek@northeastern.edu

Contents

1 Background **3**
1.1 Protein inference assisted by peptide abundance . . . 3

2 Methods **3**
2.1 Additional implementation details . . . 3

3 Case studies **5**
3.1 Additional details on Case study 1: Protein degrader . . . 5
3.2 Simulated and resampled datasets . . . 6

4 Results **8**
4.1 Precision of parameter estimation . . . 8
4.2 Modeling the contribution of shared peptides improved the bias and variance of log_2-fold change estimation, in particular with few unique peptides . . . 8
4.3 Modeling the contribution of shared peptides reduced the number of reported sites in the PTM study . . . 10
4.4 Weighted summarization improved sensitivity and specificity of detecting differentially abundant proteins . . . 11
4.5 A non-informative initialization of the algorithm was preferred for clusters where some proteins were only identified by shared peptides . . . 12
4.6 Weighted summarization enabled the quantification of a protein that was only identified by shared peptides . . . 13
4.7 Robust estimation reduced the MSE of $\log_2$-fold change estimation relative protein quantification compared to least-squares . . . 14

1 Background

1.1 Protein inference assisted by peptide abundance

This section describes existing methods for protein inference or quantification that use peptide-levels information (in particular quantitative profiles) to aid the task.

Quantifere (Lukasse and America, 2014) aims to increase protein coverage beyond what would be otherwise retained by exclusion or parsimony. In this approach, proteins included in parsimony inference results are called primary proteins. To discover additional secondary proteins, peptide profiles are grouped by their correlation. Secondary proteins are identified by peptides that do not correlate with peptides of the primary proteins.

PeCorA (Peptide Correlation Analysis, (Dermit *et al.*, 2020)) compares the quantitative profiles of peptides that match a same protein, and finds outlying peptides. While the original implementation was intended for uniquely matched peptides, it is in principle applicable to proteins with shared peptides, with outlying profiles forming separate protein groups.

COPF (COrrelation-based functional ProteoForm assessment, (Bludau *et al.*, 2021)) defines a functional proteoform group, i.e. a group of peptides that are both derived from a same gene and have co-varying abundance profiles. The peptide profiles are clustered by their pairwise correlation, and segmented with hierarchical clustering. Finally, the peptide clusters are evaluated with a scoring statistic, quantifying the difference in between- and within-cluster correlations.

VIQoR (Tsiamis and Schwämmle, 2022) is an iterative approach that offers not only protein inference, but also detection of differential protein abundance. At the protein inference step, VIQoR reports all possible protein memberships of a peptide, a parsimony result, or a soft parsimony result. With soft parsimony, a protein with the highest number of matching peptides in a cluster is grouped with its subsets and is considered inferred. In other words, it infers groups of subset proteins rather than individual proteins. The procedure is repeated until at least one protein explains the presence of every peptide. Next, it calculates peptide-level fold-changes, and uses factor analysis and fast-FARMS algorithm (Zhang *et al.*, 2017) to estimate protein weights for each peptide. Finally, weighted peptide-level estimates are combined into a single protein-level (log-)fold change.

2 Methods

2.1 Additional implementation details

Algorithm 1 describes iterative estimation procedure for the proposed model. While the notation used in article helps making the connection to the established MSstatsTMT summarization, here we introduce an equivalent vector notation that simplifies the description of parameter estimation. In Step 1, the algorithm estimates the initial values of protein-level summaries, usually based on unique peptides. Then, Step 2 estimates the parameters $Weights_{fk}$ using the estimates in Step 1. Once these starting values are estimated, the algorithm iterates between Steps 3 and 4 until convergence of weights. Step 3 is equivalent to fitting a linear model where weights $Weight_{fk}$ are values of variables associated with parameters $Channel_{kc}$ and $Protein_k$. Step 4 is equivalent to fitting a linear model where weights $Weight_{fk}$ are treated as unknown parameters and known values of $Channel_{kc}$ and $Protein_k$ populate the respective variables.

Algorithm 1: Iterative parameter estimation

Input: $V(f), f = 1, \ldots, F$ - peptide membership sets
$X_{f,c}$ - observed intensities of features $f = 1, \ldots, F$ in channels $c = 1, \ldots, C$
tol - tolerance

Output: $\hat{Y}_{kc}, c = 1, \ldots, C$ estimated profiles of proteins $k = 1, \ldots, K$.

Define: $\mathbf{P} = (Protein_k)_{k=1,\ldots,K}$, $\mathbf{D} = (Channel_{kc})_{k=1,\ldots,K,c=1,\ldots,C}$, $\mathbf{G} = (Feature_f)_{f=1,\ldots,F}$, $\mathbf{W} = (Weight_{fk})_{f=1,\ldots,F,k=1,\ldots,K}$

Initialize $i = 0$, $\widehat{\mathbf{P}}^{(0)}, \widehat{\mathbf{D}}^{(0)}$ [e.g. by averaging the intensities of the unique features]
$\widehat{\mathbf{W}}^{(0)} \leftarrow \arg\min_{\mathbf{W},\mu,\mathbf{G}} \tilde{L}\left(\mathbf{W}, \mu, \mathbf{G} | \widehat{\mathbf{P}}^{(0)}, \widehat{\mathbf{D}}^{(0)}\right)$
while $\sum_{f,k} |W_{fk}^{(i+1)} - W_{fk}^{(i)}| > tol$ **do**
    $\hat{\mathbf{D}}^{(i+1)}, \hat{\mathbf{P}}^{(i+1)}, \mu, \mathbf{G} \leftarrow \arg\min \tilde{L}_{\mathbf{P},\mathbf{D},\mu,\mathbf{G}}\left(\mathbf{P}, \mathbf{D}, \mu, \mathbf{G} | \widehat{W}^{(i)}\right)$
    $\widehat{\mathbf{W}}^{(i+i)}, \mathbf{G} \leftarrow \arg\min_{\mathbf{W},\mathbf{G}} \tilde{L}\left(\mathbf{W}, \mathbf{G} | \widehat{\mathbf{P}}^{(i+1)}, \widehat{\mathbf{D}}^{(i+1)}\right)$
    $i \leftarrow i + 1$
end
Output: $\hat{Y}_{kc} = \hat{\mu} + \widehat{P}_k + \widehat{D}_{kc}, k = 1, \ldots, K, c = 1, \ldots, C$

3 Case studies

3.1 Additional details on Case study 1: Protein degrader

This section contains additional details regarding this previously unpublished dataset

Sample Preparation EOL-1 cells were treated with either DMSO or GNE-0011 for 0, 30, 60, 120, or 480 minutes and pelleted before proteomics sample preparation. Cell pellets were lysed in a buffer containing 8 M urea, 50 mM HEPES pH 8.0, 150 mM NaCl, benzonase nuclease (Sigma Aldrich, 1 µL/10 mL lysis buffer), and one cOmplete Protease inhibitor cocktail (Roche). Protein concentrations were determined by BCA assay (ThermoFisher Pierce) and an equal amount of protein was taken for further sample preparation. Each sample was reduced by the addition of 5 mM dithiothreitol (DTT) at 37° C for 45 minutes (min), alkylated by the addition of 15 mM iodoacetamide at room temperature (RT) for 30 min, and capped by the addition of 5 mM DTT at RT for 15 min. Proteins were then digested by the addition of Lys-C (Wako) at a substrate:enzyme ratio of 50:1 for 3 hours at 37° C. Samples were then diluted to 2 M urea with a buffer of 50 mM HEPES pH 8.5 and further digested with Trypsin (Promega) at a substrate:enzyme ratio of 50:1 at RT overnight. Resulting peptides were then acidified and desalted via Sep-Pak (Waters) and dried via vacuum centrifugation.

For TMT labeling samples were resuspended in 200 mM HEPES pH 8.5 and labeled with TMT-10plex reagents (ThermoFisher Pierce) according to the manufactures instructions. Labeled peptides were mixed together to ensure equal protein loading across samples and dried before fractionation. The multiplexed sample was then fractionated by offline liquid chromatography using an Agilent 1100 series HPLC system. Dried peptides were resuspended in water and loaded onto an Agilent Zorbax 300 Extend C-18 analytical column (2.1 x 150 mm and 3.5 μ m particle size). Solvent A consisted of 25 mM Ammonium Formate (pH 9.7) while solvent B was 100% MeCN. Peptides were collected into 96 fractions after which they were concatenated into 24 fractions using a checkerboard pattern, 12 fractions were selected for mass spectrometry analysis.

Data acquisition Data were collected on an Orbitrap Fusion Lumos Mass Spectrometer (ThermoFisher Scientific) coupled to an RSLCnano U3000 liquid chromatography system (ThermoFisher Scientific). Peptides were loaded onto a New Objective PicoFrit column packed with Acquity BEH C18 material (1.7 μm, 100 μm x 250 mm) at a flow rate of 800 nL/min in 98% solvent A (98% water/2% acetonitrile/0.1% formic acid). Peptides were then separated at a flow rate of 450 nL/min using a gradient of 2% to 30% Solvent B (98% acetonitrile/2% water/0.1% formic acid) over 140 min. The total run time for each fraction was 180 min.

Synchronous precursor selection (SPS) MS3 data analysis was utilized to improve accuracy of resulting quantification. Intact peptides were detected through Orbitrap MS1 analysis (120,000 resolution, scan range 350-1350, max injection time 50 ms, AGC target 1,000,000) and the top 10 precursors were selected for MS2 analysis excluding previously picked precursors for 45 seconds (10 ppm mass tolerance, selection of one precursor per protein). Peptides were isolated (0.5 Th isolation window, fragmented (CID, normalized collision energy or 35) and analyzed in the ion trap (Turbo scan, max injection time 100 ms, AGC target 20,000). Following MS2 analysis the top 8 peaks were selected for MS3 quantitative analysis using charge dependent isolation widths of 1.2 (z = 2), 0.8 (z=3), 0.5 (z >= 4) but common fragmentation parameters (HCD, normalized collision energy 55, AGC target 250,000, max injection time 150 ms, 50,000 resolution). To improve accuracy only fragment ions within 400-2000 Th were selected and fragment ions were excluded -50 Th below and 5 Th above the precursor mass as well as within the isobaric tag loss region for TMT-10plex. Raw mass spectrometry data were deposited into the MassIVE repository (ID: MSV000094252, reviewer Password : BRD4Degrader).

Spectral processing MS/MS spectra for the BRD dataset was searched using the Comet search algorithm (version 2017.01) against a concatenated target/decoy database comprised of the Swissprot human protein sequences (version 2017.08), contaminants and the reversed decoy versions of each sequence. For all datasets, a 50 ppm precursor ion mass tolerance and a 1.0005 fragment bin tolerance were selected with tryptic specificity up to two missed cleavages. Fixed modifications of carbamidomethyl on cysteine residues (+57.0215) as

well as TMT 10-plex on Lysine and the peptide N-term (+229.1629). For variable modifications, methionine oxidation (+15.9949) as well as TMT 10-plex on tyrosine (+229.1629) were used. After MS/MS spectra were searched, the PSMs were filtered to a 1% peptide FDR at the search level using linear discriminant analysis (LDA) (Kirkpatrick *et al.*, 2013) Next, PSM data across all fractions were aggregated and these results were subsequently filtered to a 2% protein FDR. Finally, reporter ion intensity values were determined for each dataset using the Mojave algorithm (Zhuang *et al.*, 2013) with an isolation width of 0.5.

Evaluation metrics Let us consider k-th Time ($k = 1, \ldots, 5$) and i-th protein among BRD2, BRD3, BRD4 and two sets of $\log_2$-fold changes calculated with respect to the control group. Denoting the $\log_2$-fold change calculated based on j-th subset of unique peptides by $\mathrm{LF}_{i,t,j}$ and the $\log_2$-fold change calculated based on all available unique peptides in the same setting by $\mathrm{G}_{i,t}$, MSE of $\log_2$-fold change estimation based on j-th subset is defined as $\mathrm{MSE}_j = \frac{1}{15}\sum_{i=1}^{3}\sum_{k=1}^{5}(\mathrm{LF}_{i,k,j} - \mathrm{G}_{i,k})^2$. The results will typically present the distribution of either $\mathbf{LF}_{i,k,j}$ or MSE_j over subsets $j, j = 1, \ldots, 100$.

3.2 Simulated and resampled datasets

In computer simulations, a cluster of 5 proteins was generated. All conditions and biological replicates did not require further normalization. Protein-level data were simulated from the model assumed by MSstatsTMT:

$$\begin{aligned} Y_{mtgb} &= \mu + Mixture_m + TechRep(Mixture)_{t(m)} \\ &+ Condition_g + Subject_{mgb} + \varepsilon_{mtgb} \end{aligned} \tag{1}$$

where

$$TechRep(Mixture)_{t(m)} \overset{iid}{\sim} \mathcal{N}(0, \sigma_T^2), Subject_{mgb} \overset{iid}{\sim} \mathcal{N}(0, \sigma_S^2),$$
$$\varepsilon_{mtgb} \overset{iid}{\sim} \mathcal{N}(0, \sigma^2), \sum_g Condition_g = 0.$$

We set $\sigma^2 = \sigma_S^2 = 0.01$ and $\mu = 15$. Contrasts of interest compared each of the Conditions 2, 3, 4, 5 to Condition 1. Differences between conditions were chosen such that true $\log_2$-fold changes were equal to 0, -0.1, -0.2, -0.3, -0.5, -0.7, -0.75, -1, -1.25, -1.5. Example protein-level profile plots are shown in Figure 1.

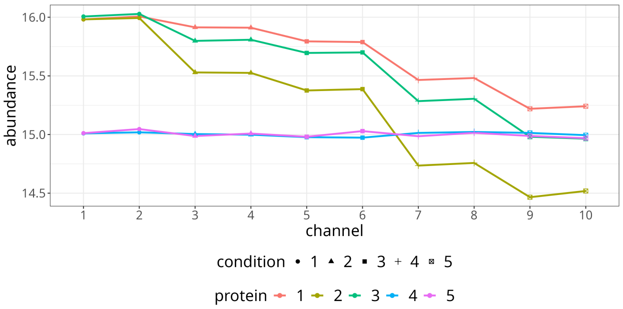

Figure 1: **Simulated data**: protein-level profiles.

Peptide-level data were generated from the proposed model. Each pair of proteins shared a fixed number of peptides with fixed weights. A different set of weights was chosen for each pair of proteins. Since for two proteins $W_{f2} = 1 - W_{f1}$, it is enough to list the first weight for each pair. The weights were set to 0.7, 0.8, 0.5, 0.6, 0.9, 0.2, 0.3, 0.5, 0.4, 0.1.

We varied the following parameters:

- standard deviation of feature-level random error: 0.1, 0.2,
- number of biological replicates: 1, 2, 3,

- number of unique peptides per protein: 1, 2, 3, 5, 10,
- number of shared peptides per pair of proteins: 3, 5, 10.

50 replicates were used for each parameter configuration.

We evalute the results in terms of mean-squared error of log-fold change estimation, sensitivity and specificity. Mean-squared error was defined as an average of squared differences between true and estimated $\log_2$-fold changes across all comparisons in each repetition of the simulation for each combination of parameters. Similarly, sensitivity was defined as a proportion of rejected hypotheses among true hypotheses across all comparisons in each iteration for each combination of parameters, and specificity was defined as the ratio of non-rejected hypotheses to true null hypotheses across all comparisons in each repetition for each combination of parrameters.

4 Results

4.1 Precision of parameter estimation

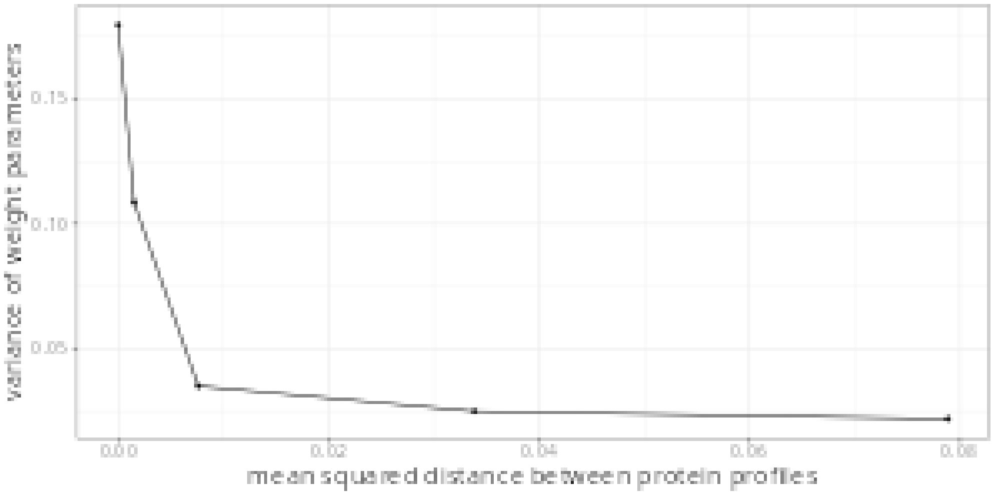

Figure 2: **Simulated data: diversity of protein profiles in a cluster impacted the variance of the estimated weights**. Weight variances were estimated with L_2 norm on a data set simulated from the proposed model with 2 proteins, 2 unique peptides per protein, and 5 shared peptides.

Figure (2) illustrates the relationship between variance of weights estimation and strength of differences between profiles of proteins on a simulated protein cluster with varying diversity of protein profiles. As the diversity between quantitative profiles at the protein level becomes more pronounced, the variance of the estimated weights decreases (and the precision increases). Hence, the estimated weights are most meaningful when proteins in a cluster exhibit clear and varied quantitative profiles.

4.2 Modeling the contribution of shared peptides improved the bias and variance of log_2-fold change estimation, in particular with few unique peptides

Protein degrader study Figure 3 presents the difference in mean-squared error of $\log_2$-fold change estimation between the proposed approach and alternative approaches as a function of the number of unique peptides per protein based on the Protein degrader Case study resampling. We used the same resampling method as in the Section 5.3 of the main manuscript. The overall quality of estimation improved substantially with the increase in the number of unique peptides. The difference in fold-change estimation error was more pronounced at very low unique peptides counts. This is in line with the results presented in the main manuscript.

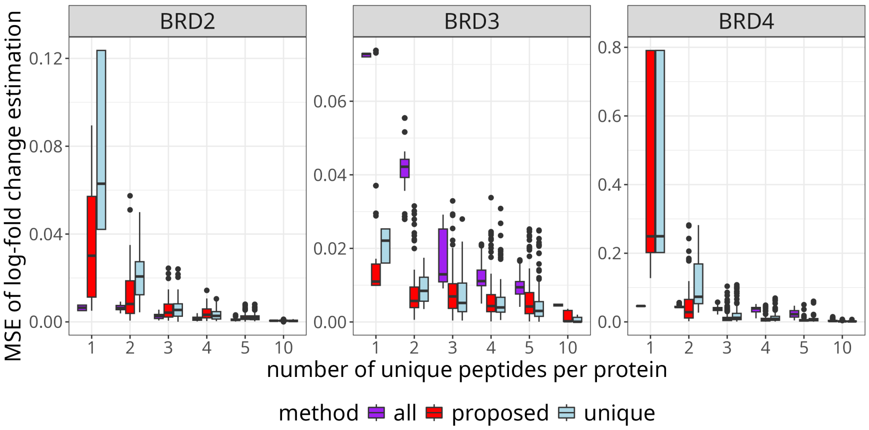

Figure 3: **Resampling simulation based on protein degrader study BRD cluster: modeling the contribution of shared peptides improved the estimation of $\log_2$-fold change as compared to using all or unique peptides.**

Since it is common in the literature to recommend protein quantification with at least two unique peptides, we evaluated the proposed approach more specifically in presence of 1, 2 and 3 unique peptides per protein and all available shared peptides. Figure 4 summarizes results of the estimation. Figure 5 (a) presents results of an alternative resampling simulation, where each protein had a single unique peptide. The proposed approach reduced both the bias and the variance of the estimation as compared to the other approaches. Figure 5 (b) provides example profiles plots of subsets of noisy unique peptides.

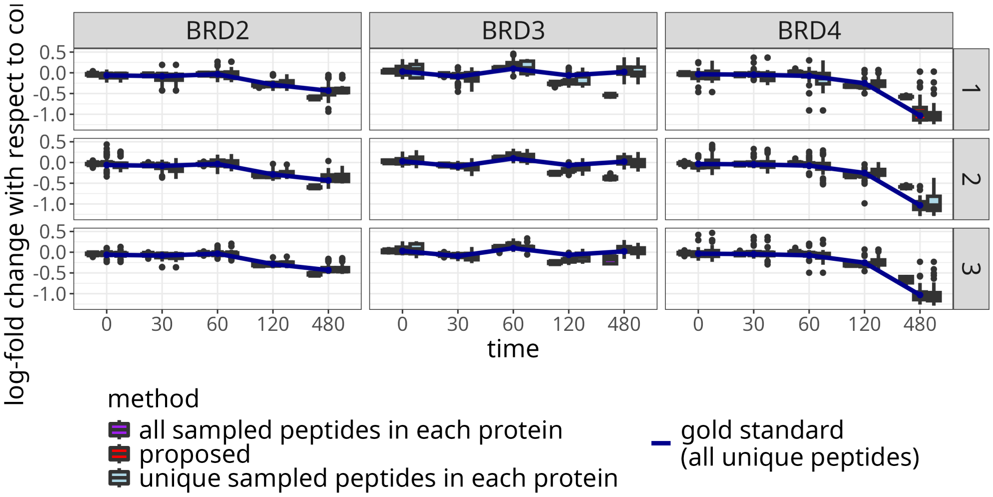

Figure 4: **Resampling simulation based on protein degrader study BRD cluster: the proposed approach improved $\log_2$-fold change estimation**.

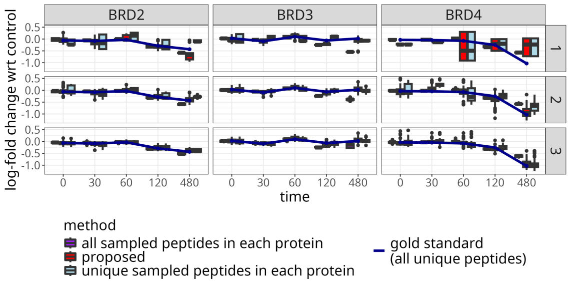

(a)

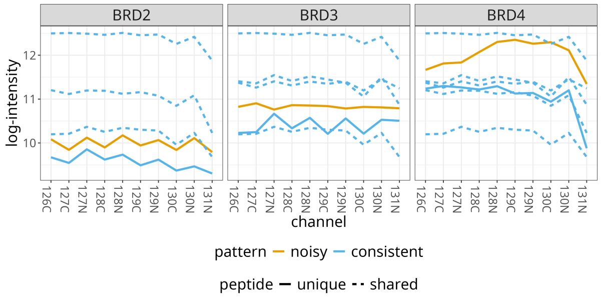

(b)

Figure 5: **Resampling simulation based on protein degrader study BRD cluster**. (a) The proposed approach improved the estimation of protein summaries in presence of outliers. (b) Example profile plots of the resampling simulation that included an outlying peptide for each protein.

Computer simulations. We further investigated the accuracy of $\log_2$-fold change estimation by computer simulation described in Section 3.2. Figure 6 presents mean-squared error of the estimation as a function of a proportion of unique peptides. The improvement of the estimation by proposed approach as compared to the estimation that used either all or unique peptides was once again most pronounced at lower proportions of unique peptides per protein.

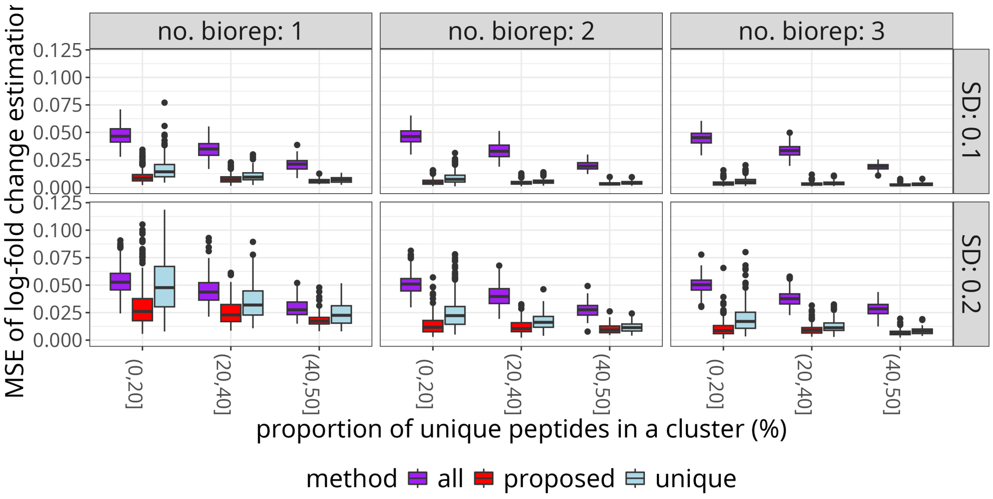

Figure 6: **Computer simulation: Modeling the contribution of shared peptides improved the bias and the variance of $\log_2$-fold change estimation**. Distribution of MSE of $\log_2$-fold change estimation across 50 replicates of the simulation study described in Section 3.2.

4.3 Modeling the contribution of shared peptides reduced the number of reported sites in the PTM study

Table 1 compares the $\log_2$-fold changes estimated by the proposed approach and the concatenation approach. The $\log_2$-fold changes estimated for the concatenated site in the original analysis were similar to the values estimated by the proposed approach for the site S236 for most comparisons. The proposed approach did not create the additional concatenated site.

Protein	Label	log_2-fold change		SE	
		concat.	proposed	concat.	proposed
E9Q6J5_S236	Infected-Uninfected	2.59	2.86	0.23	0.20
E9Q6J5_S236	KO-WT	-0.28	-0.70	0.21	0.18
E9Q6J5_S236	KO_Early-KO_Uninfected	3.30	3.74	0.37	0.31
E9Q6J5_S236	KO_Early-WT_Early	-0.07	-0.43	0.34	0.29
E9Q6J5_S236	KO_Late-KO_Uninfected	2.45	2.53	0.37	0.31
E9Q6J5_S236	KO_Late-WT_Late	-0.12	-0.60	0.34	0.29
E9Q6J5_S236	KO_Uninfected-WT_Uninfected	-0.67	-1.07	0.40	0.34
E9Q6J5_S236	WT_Early-Wt_Uninfected	2.71	3.10	0.37	0.31
E9Q6J5_S236	WT_Late-Wt_Uninfected	1.90	2.06	0.37	0.31
E9Q6J5_S236_S240	Infected-Uninfected	1.96		0.26	
E9Q6J5_S236_S240	KO-WT	-0.35		0.23	
E9Q6J5_S236_S240	KO_Early-KO_Uninfected	2.82		0.44	
E9Q6J5_S236_S240	KO_Early-WT_Early	-0.18		0.36	
E9Q6J5_S236_S240	KO_Late-KO_Uninfected	1.13		0.44	
E9Q6J5_S236_S240	KO_Late-WT_Late	-0.49		0.36	
E9Q6J5_S236_S240	KO_Uninfected-WT_Uninfected	-0.37		0.44	
E9Q6J5_S236_S240	WT_Early-Wt_Uninfected	2.64		0.37	
E9Q6J5_S236_S240	WT_Late-Wt_Uninfected	1.26		0.37	
E9Q6J5_S240	Infected-Uninfected	0.59	0.75	0.14	0.13
E9Q6J5_S240	KO-WT	0.08	-0.21	0.13	0.12
E9Q6J5_S240	KO_Early-KO_Uninfected	0.28	0.47	0.23	0.21
E9Q6J5_S240	KO_Early-WT_Early	0.03	-0.23	0.21	0.20
E9Q6J5_S240	KO_Late-KO_Uninfected	0.72	0.79	0.23	0.21
E9Q6J5_S240	KO_Late-WT_Late	0.00	-0.34	0.21	0.20
E9Q6J5_S240	KO_Uninfected-WT_Uninfected	0.19	-0.05	0.24	0.23
E9Q6J5_S240	WT_Early-Wt_Uninfected	0.44	0.66	0.23	0.21
E9Q6J5_S240	WT_Late-Wt_Uninfected	0.91	1.08	0.23	0.21

Table 1: **Multi-site PTM study: modeling the contribution of shared peptides reduced the number of reported sites without changing the majority of the quantitative results.**

4.4 Weighted summarization improved sensitivity and specificity of detecting differentially abundant proteins

Figure 7 details the results for the P16591, P16591-2, and P16591-3 cluster. Protein P16591-3 is a known interactor, present both in the the KinHub database and in the list of differentially abundant proteins in the OnePot portion of the study. The few peptides uniquely characterizing P16591-3 included flat profiles, while shared peptides exhibited a consistent pattern which enabled confirmation of a difference between conditions. Test for differential abundant based on the proposed summarization detected this protein as differentially abundant despite a relatively small $\log_2$-fold change of 1.65. Test based on summarization with unique peptides resulted in a smaller $\log_2$-fold change of -0.03, and the protein was not detected as differentially abundant. Similarly, test based on summarization with all peptides resulted in a $\log_2$-fold change of 0.17 and a conclusion of no differential abundance. Due to the small number of unique peptides, summary based on them was more sensitive to flatter, likely outlying, profiles present among them. Shared peptides exhibited consistent patterns.

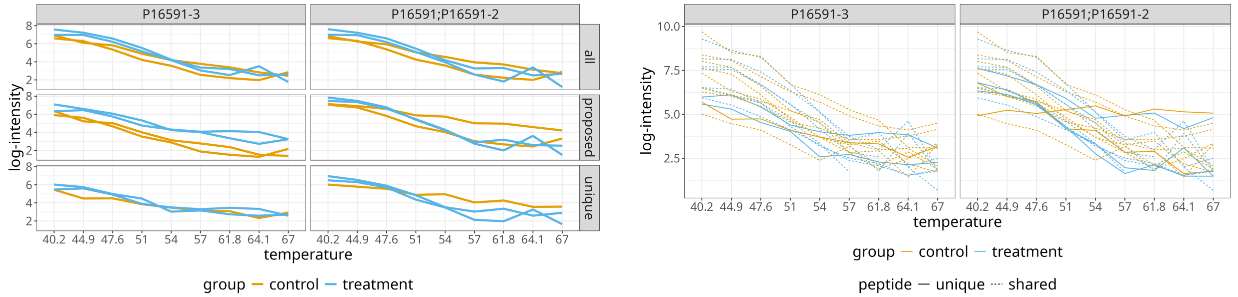

(a) (b)

Figure 7: **Thermal profiling: The proposed approach detected the differential abundance of a known interactor in the P16591, P16591-2, and P16591-3 cluster**. Test for differential abundance based on the proposed summarization detected the differential abundance of known protein interactor P16591-3, while tests based on summarization with unique peptides and all the peptides did not. (a) Protein-level summaries for each protein in each run. (b) Feature-level data including flat, outlying profiles in unique peptides.

4.5 A non-informative initialization of the algorithm was preferred for clusters where some proteins were only identified by shared peptides

Protein degrader study We considered three possible starting values for protein-level summaries: based on unique peptides, flat profiles equal to median of peptide-level intensities in a channel, and a combination of the two which uses flat profiles for subset proteins and unique peptides-based profiles for other proteins. We conducted a resampling simulation based on the BRD cluster in which for each protein we sampled all the shared peptides and 1, 2 or 3 unique peptides. Figure 8 shows that in 100 instances of the simulation with clusters where unique peptides were present for every protein, different starting points resulted in very similar $\log_2$-fold changes and mean-squared errors.

When some proteins were not identified by any unique peptides, the choice of a starting point for the fitting algorithm (initialization - Step 1 of Algorithm 1) became important. Figure 9 presents the results of 100 instances of another resampling-based simulation study. In each instance, unique peptides of one of the proteins of the BRD cluster were removed from the analysis, making it a subset protein. Then, we evaluated

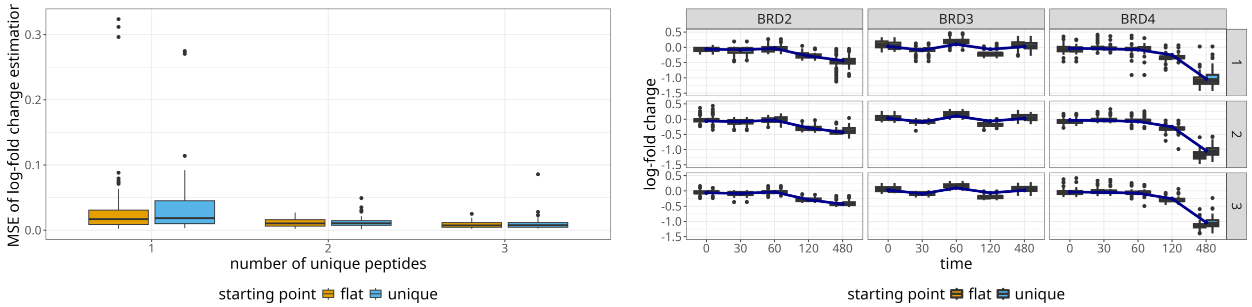

(a) (b)

Figure 8: **Resampling simulation, based on protein degrader study BRD cluster: in clusters where each protein was identified by unique peptides, we observed no substantial differences in summarization for different starting points of the algorithm.** (a) Mean-squared error of $\log_2$-fold change estimation. (b) Estimated $\log_2$-fold changes based on resampling-based simulation evaluate with different starting points of the proposed approach. *flat* refers to starting point equal to median of all observed intensities in channel (constant profile), while `unique` refers to a starting point based on unique peptides.

the ability of the proposed approach to recover the true quantitative pattern based on the shared peptides only. A flat, non-informative, starting point for all the available proteins lead to better $\log_2$-fold change estimation than using informative starting points based on the unique peptides. Hence, summarization based on the flat starting point performed well both with proteins that had unique peptides, and with subset proteins (Figure 8).

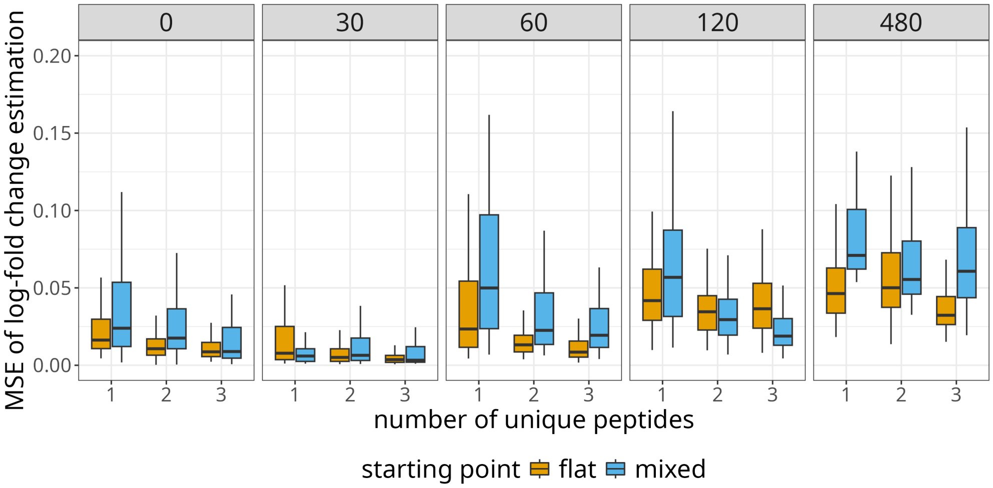

Figure 9: **Resampling simulation, based on protein degrader study BRD cluster: in clusters where some proteins were not identified by any unique peptides, flat algorithm initialization optimized protein-level summarization**. *Flat* denotes an initialization with a constant profile equal to the median of all peptide intensities for each protein, while *mixed* denotes initialization with unique peptides-based profiles whenever possible, and with flat profiles for proteins identified by shared peptides only.

4.6 Weighted summarization enabled the quantification of a protein that was only identified by shared peptides

Protein degrader study Protein summarization based on unique peptides lacks the ability to quantify proteins identified by shared peptides only. In order to evaluate the proposed approach in such scenario we conducted a resampling simulation. In each repetition of the simulation, we selected all shared peptides and 2 unique peptides for proteins BRD2 and BRD3, with no unique peptides for protein BRD4. Figure 10 shows that in the case of the protein degrader Case study presence of BRD4 protein that was only identified by shared peptides did not negatively affect the $\log_2$-fold change estimation of BRD2 and BRD3 proteins. Although the proposed approach had a higher variance of $\log_2$-fold change estimation for BRD4 protein as compared to using all the peptides, it had a higher precision.

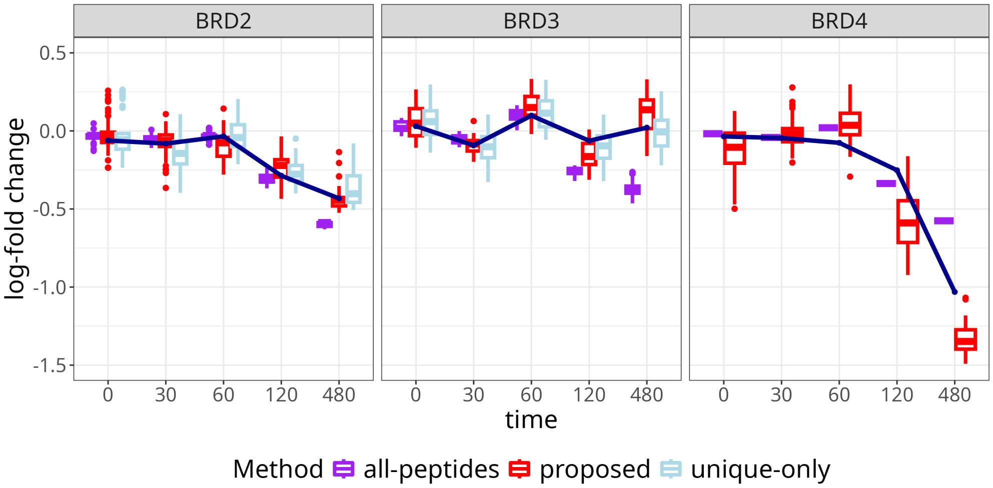

Figure 10: **Resampling simulation based on protein degrader study BRD cluster: weighted summarization enabled the quantification of a protein that was only identified by shared peptides**. Dark-blue line: estimation based on all the originally available unique peptides. Despite the lack of unique peptides, $\log_2$-fold change estimated at the final time point was close to the gold standard.

4.7 Robust estimation reduced the MSE of $\log_2$-fold change estimation relative protein quantification compared to least-squares

Protein degrader study Figure 11 compares the MSE of $\log_2$-fold change estimation for loss functions with two norms: L2 norm (ordinary least squares for fixed values of $Weight$ or $Channel$ parameters) and Huber norm in presence of outliers in unique peptides. To evaluate the loss function, we used the same simulation design as in the Section 4.3 of the main manuscript. The simulation introduced outlying quantitative profiles in each repetition. The robust lost function reduced the estimation error as compared to ordinary least squares.

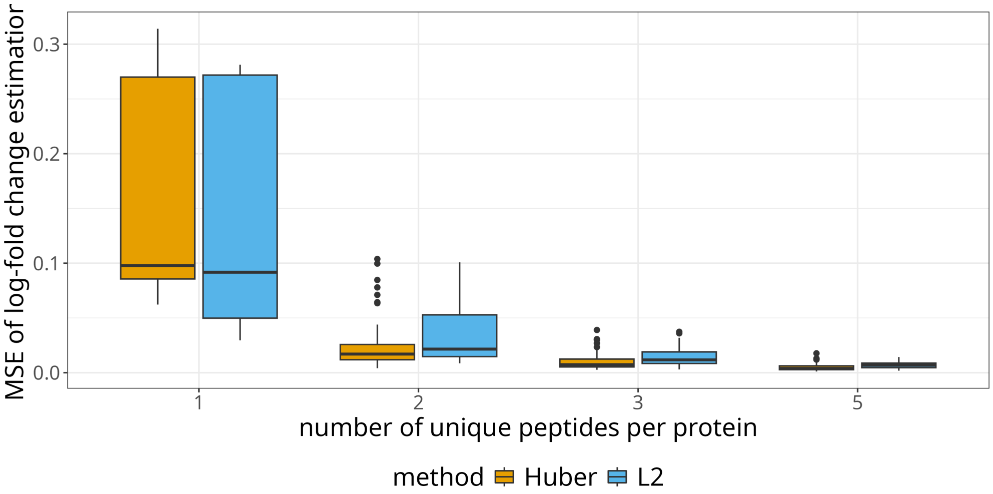

Figure 11: **Resampling simulation based on protein degrader study BRD cluster: the robust loss function reduced the MSE of $\log_2$-fold changes estimation**. MSE of $\log_2$-fold change estimation based on 100 replications of the resampling-based simulation study.

References

Bludau, I. *et al.* (2021). Systematic detection of functional proteoform groups from bottom-up proteomic datasets. *Nature Communications*, **12**, 1.

Dahl, D. B. *et al.* (2019). *xtable: Export Tables to LaTeX or HTML*. R package version 1.8-4.

Dermit, M. *et al.* (2020). Peptide correlation analysis (PeCorA) reveals differential proteoform regulation. *Journal of Proteome Research*, **20**, 1972.

Kirkpatrick, D. S. *et al.* (2013). Phosphoproteomic characterization of DNA damage response in melanoma cells following MEK/PI3K dual inhibition. *Proceedings of the National Academy of Sciences*, **110**(48), 19426–19431.

Lukasse, P. N. and America, A. H. (2014). Protein inference using peptide quantification patterns. *Journal of Proteome Research*, **13**, 3191.

Tsiamis, V. and Schwämmle, V. (2022). VIQoR: a web service for visually supervised protein inference and protein quantification. *Bioinformatics*, **38**, 2757.

Zhang, B. *et al.* (2017). Covariation of peptide abundances accurately reflects protein concentration differences. *Molecular & Cellular Proteomics*, **16**, 936.

Zhuang, G. *et al.* (2013). Phosphoproteomic analysis implicates the mTORC2-FoxO1 axis in VEGF signaling and feedback activation of receptor tyrosine kinases. *Science Signaling*, **6**, ra25.
